# Supplementary material for: Association between gender social norms and cardiovascular disease mortality and life expectancy: an ecological study
Source: BMJ Open. 2023 Apr 27;13(4):e065486. doi: 10.1136/bmjopen-2022-065486 (PMC10151956; doi:10.1136/bmjopen-2022-065486)
Supplement: Supplementary data [file bmjopen-2022-065486supp001.pdf]

Supplemental Material:

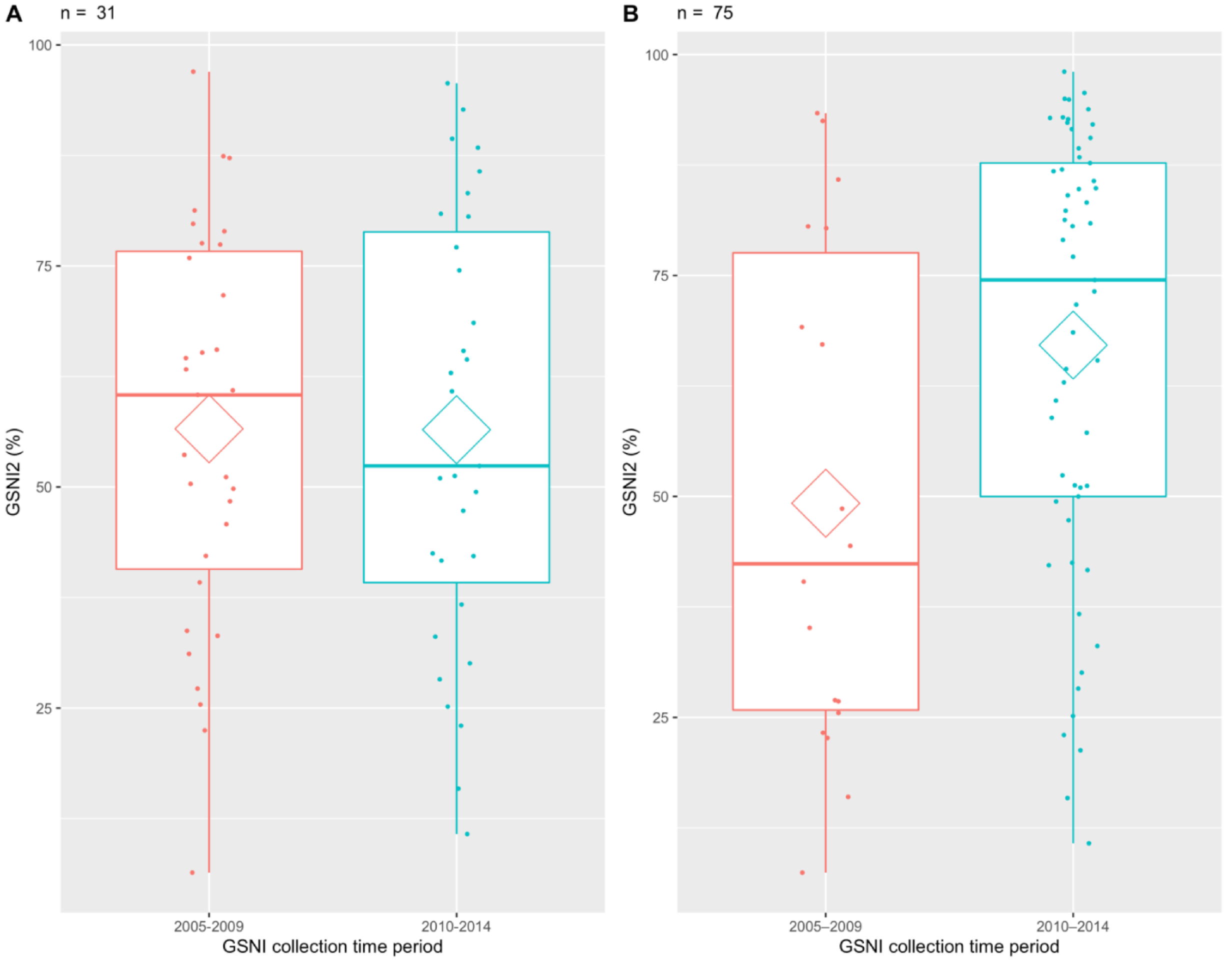

Figure S1. A. Box plots comparing GSNI2 values between countries that had values for both 2005-2009 collection period and 2010-2014 collection period. **B.** Box plots splitting the GSNI2 values included in the analysis by period of collection.

Table S1. List of countries used within scatter plots (figure 1 and 2) and univariable analyses
n=75

Algeria	India	Poland
Andorra	Indonesia	Qatar
Argentina	Iran, Islamic Republic of	Romania
Armenia	Iraq	Russian Federation
Australia	Japan	Rwanda
Azerbaijan	Jordan	Serbia
Belarus	Kazakhstan	Singapore
Brazil	Korea (Republic of)	Slovenia
Bulgaria	Kuwait	South Africa
Burkina Faso	Kyrgyzstan	Spain
Canada	Lebanon	Sweden
Chile	Libya	Switzerland
China	Malaysia	Thailand
Colombia	Mali	Trinidad and Tobago
Cyprus	Mexico	Tunisia
Ecuador	Moldova, Republic of	Turkey
Estonia	Morocco	Ukraine
Ethiopia	Netherlands	United Kingdom
Finland	New Zealand	United States
France	Nigeria	Uruguay
Georgia	Norway	Uzbekistan
Germany	Pakistan	Viet Nam
Ghana	Palestine, State of	Yemen
Haiti	Peru	Zambia
Hungary	Philippines	Zimbabwe

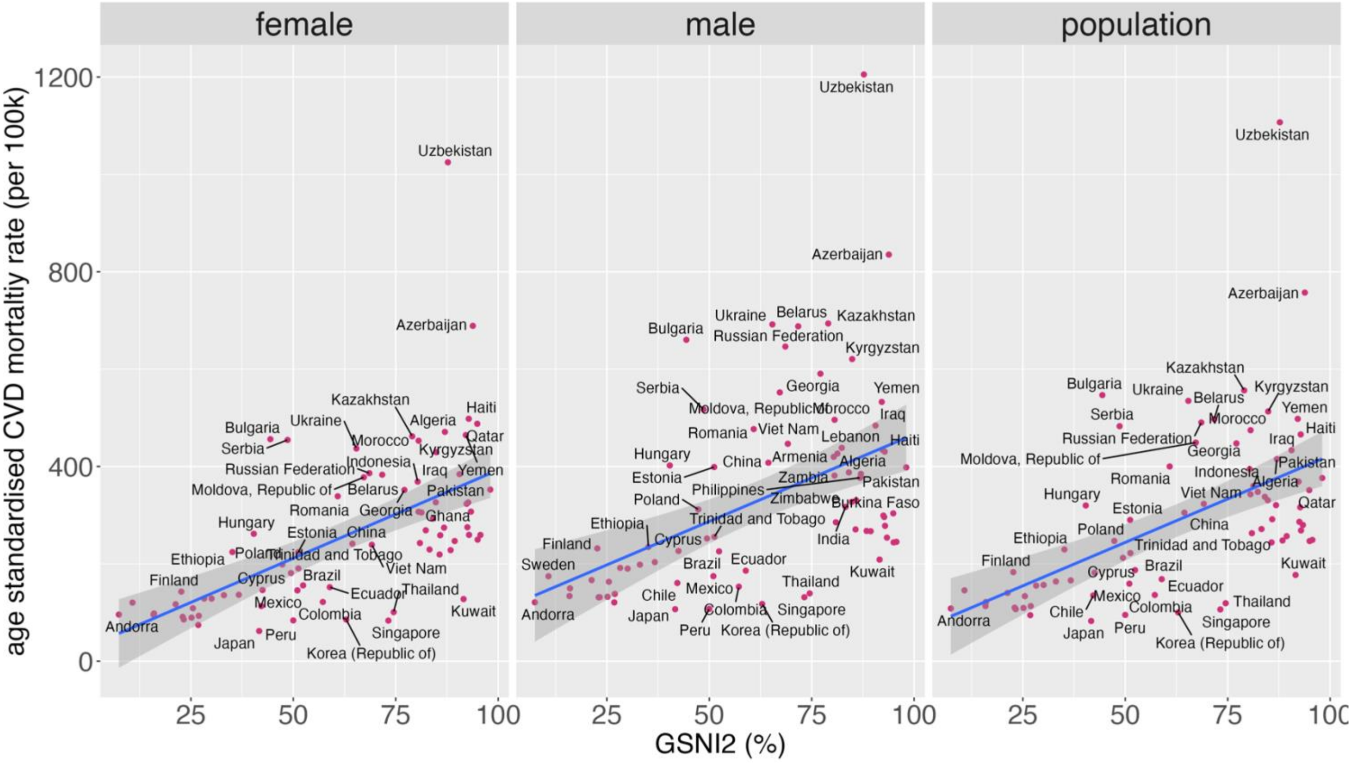

Figure S2. Scatter plots of GSNI2 against female, male and population age standardised CVD mortality rates. (GSNI2 – Gender Social Norms Index 2; CVD – Cardiovascular disease. For an interactive version of this graph please visit https://ilsumme.github.io/gsn-cvd-le/

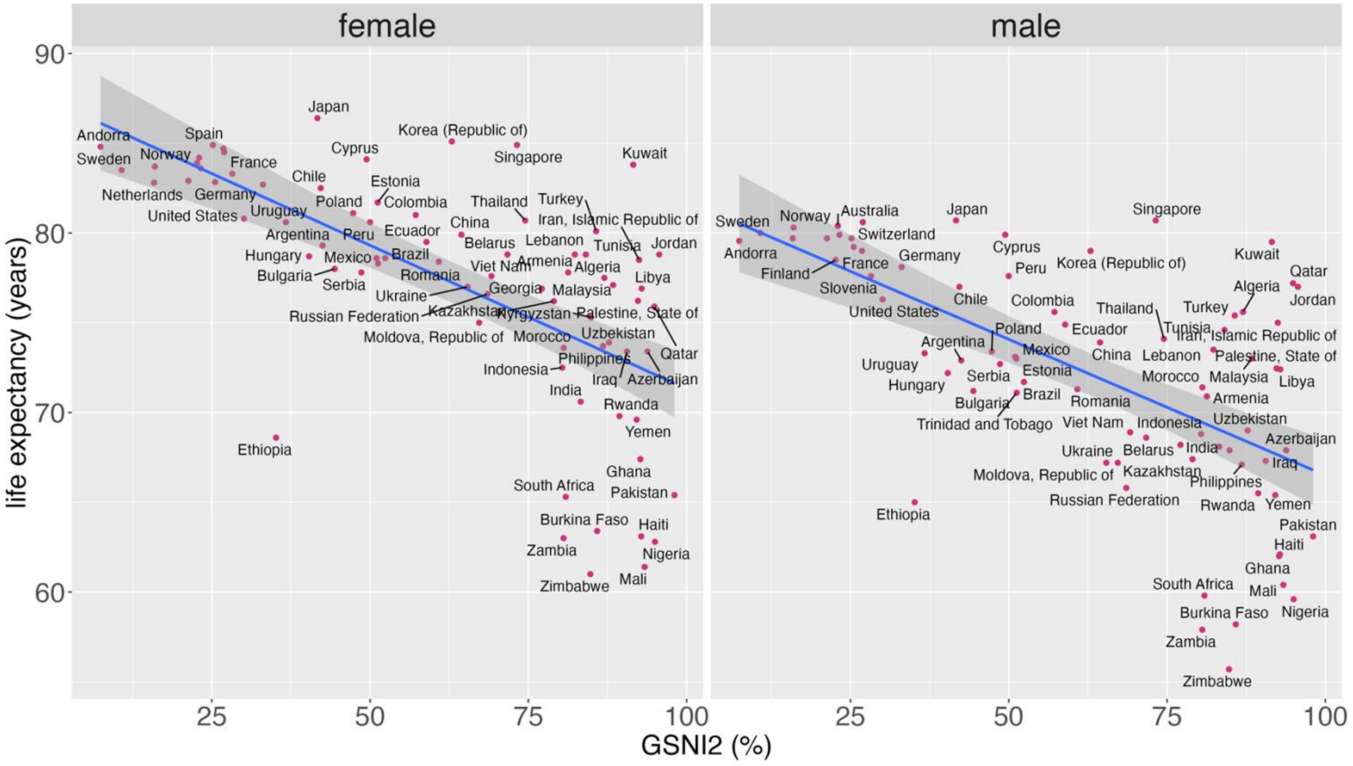

Figure S3. Scatter plots of GSNI2 against female and male life expectancy. (GSNI2 – Gender Social Norms Index 2; CVD – Cardiovascular disease. For an interactive version of this graph please visit https://ilsumme.github.io/gsn-cvd-le/

Table S2. List of countries used within multivariable analyses (Andorra not included in female life expectancy outcome as maternal mortality ratio not available; Qatar not included in CVD ratio outcome as identified as an influential outlier)
n=67

Algeria	Ghana	Philippines
Andorra	Haiti	Poland
Argentina	Hungary	Qatar
Armenia	India	Romania
Australia	Indonesia	Rwanda
Azerbaijan	Iraq	Serbia
Belarus	Japan	Singapore
Brazil	Jordan	Slovenia
Bulgaria	Kazakhstan	South Africa
Burkina Faso	Kuwait	Spain
Canada	Lebanon	Sweden
Chile	Libya	Switzerland
China	Malaysia	Thailand
Colombia	Mali	Trinidad and Tobago
Cyprus	Mexico	Tunisia
Ecuador	Morocco	Turkey
Estonia	Netherlands	Ukraine
Ethiopia	New Zealand	United Kingdom
Finland	Nigeria	United States
France	Norway	Uruguay
Georgia	Pakistan	Uzbekistan
Germany	Peru	Zambia
		Zimbabwe

	Dependent variable:			
	female CVD mortality 2017		male CVD mortality 2017	
	(1)	(2)	(3)	(4)
constant	27.10 (37.18)	-118.15 (91.70)	99.23 (50.56)	-181.55 (105.32)
GSNI2	3.57*** (0.55)	4.27*** (0.85)	3.59*** (0.74)	4.78*** (0.98)
physicians per 1000		8.39 (15.71)		39.43* (18.04)
mean years of schooling		20.53* (8.80)		29.30** (10.10)
GDP per capita		-0.002* (0.001)		-0.004*** (0.001)
GSNI data collection period 2010-2014		-90.81* (38.64)		-115.70* (44.37)
R^2	0.37	0.49	0.24	0.55
Adjusted R^2	0.36	0.45	0.23	0.51
F Statistic	42.72*** (df = 1; 73)	11.80*** (df = 5; 61)	23.38*** (df = 1; 73)	14.80*** (df = 5; 61)
				* p<0.05, **p<0.01,***p<0.001

Table S3. Results of the sensitivity analysis univariable and multivariable regression models for the female and male CVD mortality outcomes. Numbers in brackets are standard errors. GSNI2 – Gender Social Norms Index 2; GDP – Gross Domestic Product; CVD – Cardiovascular disease

	Dependent variable:			
	CVD mortality 2017		female to male CVD mortality ratio 2017	
	(1)	(2)	(3)	(4)
constant	60.45 (41.48)	-132.22 (93.98)	0.58*** (0.05)	0.92*** (0.10)
GSNI2	3.54*** (0.61)	4.36*** (0.87)	0.003*** (0.001)	0.001 (0.001)
physicians per 1000		22.02 (16.10)		-0.04* (0.02)
mean years of schooling		23.92* (9.01)		-0.01 (0.01)
GDP per capita		-0.003** (0.001)		-0.0000 (0.0000)
GSNI data collection period 2010-2014		-101.63* (39.59)		-0.03 (0.04)
R^2	0.32	0.53	0.22	0.44
Adjusted R^2	0.31	0.49	0.21	0.39
F Statistic	33.75*** (df = 1; 73)	13.60*** (df = 5; 61)	20.31*** (df = 1; 73)	9.38*** (df = 5; 60)

* p<0.05, **p<0.01,***p<0.001

Table S4. Results of the sensitivity analysis univariable and multivariable regression models for CVD mortality and female to male CVD mortaliy ratio outcomes Numbers in brackets are standard errors.
GSNI2 – Gender Social Norms Index 2; GDP – Gross Domestic Product; CVD – Cardiovascular disease

	Dependent variable:			
	female life expectancy 2019		male life expectancy 2019	
	(1)	(2)	(3)	(4)
constant	87.73*** (1.36)	60.75*** (4.23)	82.27*** (1.42)	38.63*** (5.06)
GSNI	-0.15*** (0.02)	-0.04* (0.02)	-0.15*** (0.02)	-0.02 (0.02)
log (physicians per 1000)		0.92** (0.28)		0.81* (0.36)
mean years of schooling		-0.29 (0.17)		-0.47 (0.23)
log (GDP per capita)		2.46*** (0.43)		4.28*** (0.56)
GSNI data collection period 2010-2014		1.32 (0.78)		1.12 (1.06)
MMR		-0.01*** (0.003)		
R^2	0.45	0.88	0.40	0.77
Adjusted R^2	0.44	0.87	0.39	0.75
F Statistic	58.99*** (df = 1; 73)	70.88*** (df = 6; 59)	48.54*** (df = 1; 73)	40.03*** (df = 5; 61)

* p<0.05, **p<0.01,***p<0.001

Table S5. Results of the sensitivity analysis univariable and multivariable regression models for female and male life expectancy at birth. Numbers in brackets are standard errors. GSNI2 – Gender Social Norms Index 2; GDP – Gross Domestic Product; CVD – Cardiovascular disease; MMR – Maternal mortality ratio
