# Supplementary material for: Association between gender social norms and cardiovascular disease mortality and life expectancy: an ecological study
Source: BMJ Open. 2023 Apr 27;13(4):e065486. doi: 10.1136/bmjopen-2022-065486 (PMC10151956; doi:10.1136/bmjopen-2022-065486)
Supplement: Author's manuscript [file bmjopen-2022-065486.draft_revisions.pdf]

BMJ Open is committed to open peer review. As part of this commitment we make the peer review history of every article we publish publicly available.

When an article is published we post the peer reviewers' comments and the authors' responses online. We also post the versions of the paper that were used during peer review. These are the versions that the peer review comments apply to.

The versions of the paper that follow are the versions that were submitted during the peer review process. They are not the versions of record or the final published versions. They should not be cited or distributed as the published version of this manuscript.

BMJ Open is an open access journal and the full, final, typeset and author-corrected version of record of the manuscript is available on our site with no access controls, subscription charges or pay-per-view fees (http://bmjopen.bmj.com).

If you have any questions on BMJ Open's open peer review process please email info.bmjopen@bmj.com

# The association between gender social norms and cardiovascular disease mortality and life expectancy: an ecological study

| | |
|---|---|
| Journal: | *BMJ Open* |
| Manuscript ID | bmjopen-2022-065486 |
| Article Type: | Original research |
| Date Submitted by the Author: | 10-Jun-2022 |
| Complete List of Authors: | Lyell, Iona; Lancaster University, Faculty of Health and Medicine<br>Khan, S; Northwestern University Feinberg School of Medicine<br>Limmer, Mark; Lancaster University, Faculty of Health and Medicine<br>O'Flaherty, M; University of Liverpool, Department of Public Health & Policy<br>Head, Anna; University of Liverpool, Department of Public Health & Policy |
| Keywords: | Cardiac Epidemiology < CARDIOLOGY, PUBLIC HEALTH, EPIDEMIOLOGY |
| | |

SCHOLARONE™
Manuscripts

*I, the Submitting Author has the right to grant and does grant on behalf of all authors of the Work (as defined in the below author licence), an exclusive licence and/or a non-exclusive licence for contributions from authors who are: i) UK Crown employees; ii) where BMJ has agreed a CC-BY licence shall apply, and/or iii) in accordance with the terms applicable for US Federal Government officers or employees acting as part of their official duties; on a worldwide, perpetual, irrevocable, royalty-free basis to BMJ Publishing Group Ltd ("BMJ") its licensees and where the relevant Journal is co-owned by BMJ to the co-owners of the Journal, to publish the Work in this journal and any other BMJ products and to exploit all rights, as set out in our licence.*

*The Submitting Author accepts and understands that any supply made under these terms is made by BMJ to the Submitting Author unless you are acting as an employee on behalf of your employer or a postgraduate student of an affiliated institution which is paying any applicable article publishing charge ("APC") for Open Access articles. Where the Submitting Author wishes to make the Work available on an Open Access basis (and intends to pay the relevant APC), the terms of reuse of such Open Access shall be governed by a Creative Commons licence – details of these licences and which Creative Commons licence will apply to this Work are set out in our licence referred to above.*

*Other than as permitted in any relevant BMJ Author's Self Archiving Policies, I confirm this Work has not been accepted for publication elsewhere, is not being considered for publication elsewhere and does not duplicate material already published. I confirm all authors consent to publication of this Work and authorise the granting of this licence.*

For peer review only

**Title:**

The association between gender social norms and cardiovascular disease mortality and life expectancy: an ecological study

**Authors:**

Iona Lyell, Sadiya S. Khan, Mark Limmer, Martin O'Flaherty, Anna Head

For peer review only

**Address for Correspondence:**

Anna Head

Room 3.13, Whelan Building

University of Liverpool, Liverpool, L69 3DT

United Kingdom

Tel: +44 (0) 151 795 2664

Email: anna.head@liverpool.ac.uk

**Institutions:**

Faculty of Health and Medicine, Lancaster University, Lancaster, UK

Iona Lyell

Academic Clinical Fellow in Public Health

Northwestern University, Chicago, USA

Sadiya S. Khan

Assistant Professor of Medicine (Cardiology) and Preventative Medicine (Epidemiology)



For peer review only - http://bmjopen.bmj.com/site/about/guidelines.xhtml

Faculty of Health and Medicine, Lancaster University, Lancaster, UK

Mark Limmer

Senior Lecturer

Department of Public Health, Policy and Systems, University of Liverpool, Liverpool, UK

Martin O'Flaherty

Professor in Epidemiology

Department of Public Health, Policy and Systems, University of Liverpool, Liverpool, UK

Anna Head

Doctoral Candidate

**Key words (MeSH):** epidemiology; global health; cardiovascular diseases; life expectancy; sexism

**Word count:** 3711

For peer review only



For peer review only - http://bmjopen.bmj.com/site/about/guidelines.xhtml

# Abstract

**Objective** Examine the association between country-level gender social norms and 1) cardiovascular disease mortality rates; 2) female to male cardiovascular disease mortality ratios; and 3) life expectancy.

**Design** Ecological study with the country as the unit of analysis.

**Setting** Global, country-level data.

**Participants** Global population of countries with data available on gender social norms as measured by the Gender Social Norms Index (developed by the United Nations Development Programme).

**Main outcome measures** Country-level female and male age-standardised cardiovascular disease mortality rates, population age-standardised cardiovascular disease mortality rates, female to male cardiovascular disease mortality ratios, female and male life expectancy at birth. Outcome measure data were retrieved from the WHO and the Institute for Health Metrics and Evaluation. Multivariable linear regression models were fitted to explore the relationship between gender social norms and the outcome variables.

**Results** Higher levels of biased gender social norms, as measured by the Gender Social Norms Index, were associated with higher female, male, and population cardiovascular disease mortality rates in the multivariable models ($\beta$ 4.86, 95% CIs 3.18 to 6.54; $\beta$ 5.28, 95% CIs 3.42 to 7.15; $\beta$ 4.89, 95% CIs 3.18 to 6.60), and lower female and male life expectancy ($\beta$ -0.07, 95% CIs -0.11 to -0.03; $\beta$ -0.05, 95% CIs -0.10 to -0.01). These results included adjustment within the models for potentially confounding country level factors



For peer review only - http://bmjopen.bmj.com/site/about/guidelines.xhtml

including GDP per capita, population mean years of schooling, physicians per 1000 population, year of Gender Social Norms Index data collection and maternal mortality ratio.

**Conclusions** Our analysis suggests that higher levels of biased gender social norms are associated with higher rates of population cardiovascular disease mortality and lower life expectancy for both sexes. Future research should explore this relationship further, to define its causal role and promote public health action.

For peer review only



For peer review only - http://bmjopen.bmj.com/site/about/guidelines.xhtml

**Strengths and Limitations:**

- This study is a novel exploration of the relationship between gender social norms and cardiovascular disease and life expectancy
- Potentially confounding variables were included within the analysis to take into account differences between countries, and sensitivity analysis was carried out to test the robustness of the findings
- To carry out this analysis a quantified measure of gender social norms was used, as gender social norms are a complex social phenomenon this quantification is unlikely to reflect all aspects of these norms
- This study alone cannot lead to any conclusions about a causal relationship between biased gender social norms and cardiovascular disease mortality or life expectancy

For peer review only

## Introduction

The population health benefits of gender equity are well recognised within the global health research community and reflected in development priorities including the Sustainable Development Goals[1, 2]. However, research in the area of gender social norms and health has thus far been largely focused on issues classically considered as ‘women’s health’ related (reproduction, child health and intimate partner violence[3-14]) and there is a lack of understanding of its relationship with non-communicable diseases at a population level. It is cardiovascular disease (CVD) specifically that is the leading cause of death worldwide[15] and a significant cause of premature mortality globally for both women and men[15]. Whilst there are theoretical explanations for a connection between gender social norms and CVD mortality at a population level[16], this has not yet been explored in an ecological framework with use of country level data.

Gender and the norms associated with it are considered a construct of society, influenced by but separate from biological sex[17]. A means through which power dynamics flow within societies, gender determines actions and reactions of individuals and communities and therefore acts as a social determinant of health[17, 18]. Gender social norms are the ‘unspoken rules’ influencing how individuals enact their gender and interact with others[6, 19]. Examples of connections between gender social norms and adverse health outcomes including CVD are gender biases in treatment and research within healthcare[18, 20, 21], the influence of gender norms on the wider determinants of health including in education and employment, and influence of gender norms on CVD risk factors e.g. stress and smoking[16, 22].


For peer review only - http://bmjopen.bmj.com/site/about/guidelines.xhtml

It is theorised that increased levels of biased gender social norms may be associated with determinants and behaviours harmful to cardiovascular health on a population-level, and therefore an increased CVD burden and reduced life expectancy more broadly for both women and men[16, 23]. Therefore, this study aimed to explore the association between gender social norms, as measured by the Gender Social Norms Index and CVD mortality and life expectancy at the country level.

## Methods

This research made use of an ecological study design with the country as the unit of analysis to investigate the relationship between gender social norms and CVD mortality rates, female to male CVD mortality ratios and life expectancy.

*Outcome variables*

The outcome variables were defined as; female, male and total age-standardised CVD mortality rate per 100 000 population, female to male CVD mortality ratio, female and male life expectancy at birth in years. The CVD outcome data was derived from the Global Burden of Disease (GBD) study 2019[15]. Age-standardised rates were calculated with use of the GBD world population age standard[15], calculated from the GBD 2019 population estimates[24]. CVD as cause of death in the GBD 2019 includes 11 disease categories: ischaemic heart disease, stroke, hypertensive heart disease, atrial fibrillation/flutter, rheumatic heart disease, non-rheumatic valvular heart disease, cardiomyopathy and myocarditis, aortic aneurysm, peripheral artery disease, endocarditis and other cardiovascular and circulatory diseases[15].



For peer review only - http://bmjopen.bmj.com/site/about/guidelines.xhtml

Life expectancy at birth data was downloaded from the Global Health Observatory hosted by the WHO[25]. It is defined as ‘the average number of years that a newborn could expect to live, if he or she were to pass through life exposed to the sex and age specific death rates prevailing at the time of his or her birth, for a specific year, in a given country, territory, or geographic area.’[25] Three countries (Andorra, UK, and Palestine) did not have data available from this source, instead this data was retrieved from the Institute for Health Metrics and Evaluation[26].

*Primary explanatory variable*

The Gender Social Norms Index (GSNI) was created by the United Nations Development Programme and available to download from http://hdr.undp.org/en/gsni. It is a quantitative measure of gender social norms at the counry level and derived from the World Values Survey[27]. The data was collected in two waves of the survey undertaken between 2005-2009 and 2010-2014. The GSNI is made up of four domains (political, educational, economic and physical integrity). The share of people with at least two gender biases (GSNI2) was chosen as the primary explanatory variable. Values are provided as a percentage for each country. The value for GSNI2 can be interpreted as an estimate of the prevalence in a country of moderate to intense gender bias[28]. 75 countries have an index value available which amounts to the representation of 81% of the global population[28].

*Covariates*

GDP per capita, physicians per 1000 population and mean years of schooling were included as additional country level covariates based on evidence of the association of these variables with


For peer review only - http://bmjopen.bmj.com/site/about/guidelines.xhtml

population health[29-31]. Datasets for these variables were retrieved from The World Bank and Our World in Data[32-34]. Maternal mortality ratio was included as a covariate in the female life expectancy model and was retrieved from The World Bank[35]. It is possible that the wave of GSNI data collection may have influenced the results e.g. through passage of time or changes in methodology. Therefore, this variable was also included within the model.

*Choice of variables (year)*

Variables (both outcome and explanatory) collected for the year 2014 or closest approximation were selected as the GSNI collection period ranged from 2005-2014. This included CVD mortality, GDP per capita, physicians per 1000 population, mean years of schooling and maternal mortality ratio. Life expectancy data from 2015 was used due to availability. If a value was not available for the year of analysis for one of the explanatory variables, a value for the most recent year available was inserted. If years either side of the missing year were available, the most contemporaneous year was selected.

*Statistical analysis*

Normally distributed continuous variables were summarised using mean and standard deviation. If not normally distributed, variables were summarised using median and inter-quartile ranges. Categorical variables were summarised using frequency tables. The GSNI values were compared over time for countries with values for both collection waves, and the values with compared between collection waves. Differences in these groups were assessed visually with the use of box plots and statistically using appropriate parametric or non-parametric hypothesis tests after assessing the normality and variance of the data.

For peer review only

Scatter plots were used to visualise the relationships between each of the outcome and explanatory variables. If the relationship was not linear, consideration of variable transformation was made for further analysis.

Univariable linear regression models were computed for each of the 3 outcome variables and each of the explanatory variables in turn. Multivariable linear regression models were then created for each outcome variable using the ordinary least squares method[36]. Testing of the null hypothesis ($\beta_1 = 0$) was done with use of the $t$-distribution where $t = \beta / SE$. Where SE is the standard error of the slope. Model goodness-of-fit was assessed with use of $R^2$ and the F statistic[36, 37].

To act as a sensitivity analysis to the choice of data time points used, the analysis was repeated for outcome and covariate data collected for 2017 (2019 for life expectancy) as this is the most recent year for which the majority of data points were available.

The assumptions for linear regression models (linearity, collinearity, homoscedasticity and normality of errors) were assessed with use of appropriate plots.

The significance level for p-values was set at 0.05 for all statistical tests. The statistical assumptions required for linear regression models were tested and deemed acceptable for all models. All data management and analysis were conducted using R version 4.1.2.

*Patient and public involvement*

No members of the public or patients were involved in the design or implementation of the study.

## Results


For peer review only - http://bmjopen.bmj.com/site/about/guidelines.xhtml

Summary statistics for the 75 countries with Gender Social Norms Index values available can be viewed in *table 1*. Female age-standardised CVD mortality rate ranged from 62 per 100 000 to 1025 per 100 000 (IQR 128 to 345). Male age-standardised CVD mortality rate ranged from 107 per 100 000 to 1205 per 100 000 (IQR 180 to 429). Age-standardised CVD mortality for the total population ranged from 83 to 1107 per 100 000 (IQR 156 to 373). Japan had the lowest male, female and total CVD mortality rates and Uzbekistan the highest. The ratio of female to male CVD mortality rates ranged from 0.5 in Viet Nam to 1.6 in Qatar (IQR 0.67 to 0.85). Female life expectancy ranged from 61 to 86.4 years (IQR 74.5 to 82.6). Zimbabwe had the lowest female life expectancy and Japan the highest. Male life expectancy ranged from 55.7 years in Zimbabwe to 80.7 years in both Japan and Singapore (IQR 67.9 to 77.6).

The GSNI value for 2 or more biases ranged from 7.4% in Andorra to 98.1% in Pakistan (median 68.6, IQR 41.9 to 86.3). 31 countries had a GSNI2 value available for both collection periods, with no statistically significant difference between the values across the two time periods (paired t-test = 0.104, p-value 0.92) (*supplemental material figure S1*). Comparison was made between the GSNI2 values included in the analysis (n=75) collected for each time period (*supplemental material figure S1*). Those collected during the later period were statistically significantly higher than those collected earlier (Man-Whitney U test = 320, p-value = 0.02).

| Summary Statistics | n = 75 |
|---|---|
| | |
| **Female CVD age adjusted mortality rate (per 100 000 population)**<br>Source: Global Burden of Disease 2019 | |



For peer review only - http://bmjopen.bmj.com/site/about/guidelines.xhtml

| | |
|---|---|
| min | 62 |
| max | 1024.9 |
| median (IQR) | 241.30 (128.06, 345.30) |
| missing | 0 |
| | |
| **Male CVD age adjusted mortality rate (per 100 000 population)**<br>**Source: Global Burden of Disease 2019** | |
| min | 106.8 |
| max | 1205.2 |
| median (IQR) | 278.05 (180.32, 428.51) |
| missing | 0 |
| | |
| **Population CVD age adjusted mortality rate (per 100 000 population)**<br>**Source: Global Burden of Disease 2019** | |
| min | 82.6 |
| max | 1107 |
| median (IQR) | 262.98 (155.95, 372.59) |
| missing | 0 |
| | |
| **Ratio of female to male CVD mortality rates**<br>**Source: Global Burden of Disease 2019** | |
| min | 0.5 |
| max | 1.6 |
| median (IQR) | 0.72 (0.67, 0.85) |
| missing | 0 |
| | |
| **Female life expectancy at birth**<br>**Source: World Health Observatory** | |
| min | 61 |
| max | 86.4 |
| median (IQR) | 78.60 (74.45, 82.60) |
| missing | 0 |
| | |
| **Male life expectancy at birth**<br>**Source: World Health Observatory** | |
| min | 55.7 |
| max | 80.7 |
| median (IQR) | 73.00 (67.90, 77.60) |
| missing | 0 |
| | |
| **GSNI index 2 or more bias**<br>**Source: Gender Social Norms Index** | |


For peer review only - http://bmjopen.bmj.com/site/about/guidelines.xhtml

| | |
|---|---|
| min | 7.4 |
| max | 98.1 |
| median (IQR) | 68.56 (41.94, 86.33) |
| missing | 0 |
| | |
| **Year of Index Collection** Source: Gender Social Norms Index | |
| 2005-2009 | 18 (24) |
| 2010-2014 | 57 (76) |
| | |
| **Physicians per 1000 population** Source: World Bank | |
| min | 0 |
| max | 5 |
| median (IQR) | 71; 2.30 (1.29, 3.22) |
| missing | 4 |
| | |
| **GDP per capita** Source: World Bank | |
| min | 566.9 |
| max | 97019.2 |
| median (IQR) | 68; 9181 (4094, 31623) |
| missing | 7 |
| | |
| **Maternal mortality ratio** Source: World Bank | |
| min | 2 |
| max | 943 |
| median (IQR) | 70; 25.50 (10.25, 82.75) |
| missing | 5 |
| | |
| **Mean years of schooling** Source: Our World In Data | |
| min | 1.4 |
| max | 14 |
| median (IQR) | 69; 10.10 (7.60, 11.80) |
| missing | 6 |

*Table 1.* Summary statistics. IQR – inter-quartile range


For peer review only - http://bmjopen.bmj.com/site/about/guidelines.xhtml

GSNI2 was positively correlated with female CVD mortality rates (rho = 0.67, p-value <0.05), with male CVD mortality rates (rho 0.53, p-value <0.05) and with population CVD mortality rates (rho = 0.60, p-value <0.05). GSNI2 was also positively correlated with female to male CVD mortality ratio (rho = 0.57, p-value <0.05) (*figure 1*). GSNI2 was negatively correlated with female life expectancy (rho = -0.72, p-value <0.05) and with male life expectancy (rho = -0.61, p-value = <0.05) (*figure 2*).

75 countries had data available for the univariable regression analyses. 67 Countries had data available across all covariates for the multivariable regression analyses. Andorra was not included in the female life expectancy outcome as maternal mortality ratio not available (n = 66); Qatar was excluded from the CVD ratio outcome analysis because it was identified as an influential outlier (n = 66). The list of included countries within each analysis can be found in *supplemental material tables S1-2.*

In the univariable models, higher levels of gender bias as measured by the GSNI2 were statistically significantly associated with higher female CVD mortality rates ($\beta$ 3.63, 95% CIs 2.48 to 4.78), male CVD mortality rates ($\beta$ 3.57, 95% CIs 2.03 to 5.12) and population CVD mortality rates ($\beta$ 3.57, 95% CIs 2.29 to 4.85). The statistically significant association remained after adjusting for physicians per 1000 population, mean years of schooling, GDP per capita and GSNI data collection period (*table 2 & 3*). The multivariable model results indicate that for every 1% greater proportion of the population who hold 2 or more gender biases, the female CVD mortality rate was higher by 4.86 per 100 000 (95% CI 3.18 to 6.54), male CVD mortality rate higher by 5.28 per 100 000 (95% CI 3.42 to 7.15) and the population CVD mortality rate was higher by 4.89 per 100 000 (95% CI 3.18 to 6.60). In the univariable model GSNI2 was statistically significantly associated with female to male CVD


For peer review only - http://bmjopen.bmj.com/site/about/guidelines.xhtml

mortality ratio ($\beta$ 0.003, 95% CIs 0.002 to 0.005). This relationship was not significant within the multivariable model ($\beta$ 0.001, 95% CIs -0.001 to 0.003).

*Table 4* demonstrates the model results for the life expectancy outcomes. In the univariable analysis, higher levels of gender bias as measured by the GSNI2 were statistically significantly associated with lower female life expectancy ($\beta$ -0.16, 95% CIs -0.20 to -0.12) and lower male life expectancy ($\beta$ -0.15, 95% CIs -0.20 to -0.11). This relationship remained statistically significant after controlling for physicians per 1000 population, mean years of schooling, GDP per capita and GSNI data collection period. Maternal mortality ratio was also included within the female life expectancy model. Within the life expectancy multivariable models, the physicians per 1000 and GDP per capita variables were log transformed due to their relationship with the outcome variable. In the final life expectancy model the coefficient indicates that for every 1% increase in the GSNI2, female life expectancy was lower by 0.07 years (25.6 days) (95% CI -0.11 to -0.03) and male life expectancy was lower by 0.05 years (18.3 days) (95% CI -0.10 to -0.01).

The sensitivity analysis made use of data from 2017 and 2019 with no change in direction of association between each outcome variable and GSNI2. The only change in statistical significance compared to the primary analysis was the multivariable male life expectancy model in which male life expectancy was not found to be statistically significantly associated with GSNI2 (*supplemental material tables S3-5*).


For peer review only - http://bmjopen.bmj.com/site/about/guidelines.xhtml
For peer review only

| | *Dependent variable:* | | | |
|---|---|---|---|---|
| | female CVD mortality | | male CVD mortality | |
| | (1) | (2) | (3) | (4) |
| constant | 30.04 (39.25) | -133.15 (94.46) | 108.45* (52.87) | -184.13 (104.74) |
| GSNI2 | 3.63*** (0.58) | 4.86*** (0.84) | 3.57*** (0.78) | 5.28*** (0.93) |
| physicians per 1000 | | 32.31 (19.26) | | 70.59** (21.35) |
| mean years of schooling | | 13.75 (9.37) | | 21.71* (10.39) |
| GDP per capita | | -0.002* (0.001) | | -0.004*** (0.001) |
| GSNI data collection period 2010-2014 | | -97.89* (40.74) | | -127.18** (45.17) |
| $R^2$ | 0.35 | 0.49 | 0.23 | 0.56 |
| Adjusted $R^2$ | 0.34 | 0.44 | 0.21 | 0.53 |
| F Statistic | 39.68*** (df = 1; 73) | 11.54*** (df = 5; 61) | 21.23*** (df = 1; 73) | 15.62*** (df = 5; 61) |

* p<0.05, **p<0.01,***p<0.001

*Table 2.* Results of univariable and multivariable regression models for the female and male CVD mortality outcomes. Numbers in brackets are standard errors.

GSNI2 – Gender Social Norms Index 2; CVD – Cardiovascular disease


For peer review only - http://bmjopen.bmj.com/site/about/guidelines.xhtml

| | *Dependent variable:* | | | |
|---|---|---|---|---|
| | CVD mortality | | female to male CVD mortality ratio | |
| | (1) | (2) | (3) | (4) |
| constant | 65.58 (43.71) | -140.69 (95.92) | 0.57*** (0.05) | 0.90*** (0.09) |
| GSNI2 | 3.57*** (0.64) | 4.89*** (0.85) | 0.003*** (0.001) | 0.001 (0.001) |
| physicians per 1000 | | 47.45* (19.55) | | -0.04* (0.02) |
| mean years of schooling | | 17.25 (9.52) | | -0.01 (0.01) |
| GDP per capita | | -0.003** (0.001) | | -0.0000 (0.0000) |
| GSNI data collection period 2010-2014 | | -110.79** (41.36) | | -0.03 (0.04) |
| $R^2$ | 0.30 | 0.53 | 0.24 | 0.46 |
| Adjusted $R^2$ | 0.29 | 0.49 | 0.22 | 0.42 |
| F Statistic | 30.97*** (df = 1; 73) | 13.54*** (df = 5; 61) | 22.44*** (df = 1; 73) | 10.30*** (df = 5; 60) |

* p<0.05, **p<0.01,***p<0.001

*Table 3.* Results of univariable and multivariable regression models for CVD mortality and female to male CVD motality ratio. Numbers in brackets are standard errors.

GSNI2 – Gender Social Norms Index 2; CVD – Cardiovascular disease

For peer review only

| | *Dependent variable:* | | | |
|---|---|---|---|---|
| | female life expectancy | | male life expectancy | |
| | (1) | (2) | (3) | (4) |
| constant | 87.31*** (1.46) | 68.74*** (4.82) | 81.65*** (1.51) | 50.48*** (5.98) |
| GSNI | -0.16*** (0.02) | -0.07*** (0.02) | -0.15*** (0.02) | -0.05* (0.02) |
| log (physicians per 1000) | | 1.27* (0.61) | | 1.95** (0.66) |
| mean years of schooling | | -0.41 (0.21) | | -0.69* (0.27) |
| log (GDP per capita) | | 1.82*** (0.46) | | 3.29*** (0.58) |
| GSNI data collection period 2010-2014 | | 1.30 (0.91) | | 0.95 (1.14) |
| MMR | | -0.01*** (0.003) | | |
| $R^2$ | 0.43 | 0.86 | 0.39 | 0.76 |
| Adjusted $R^2$ | 0.42 | 0.84 | 0.38 | 0.74 |
| F Statistic | 55.39*** (df = 1; 73) | 60.02*** (df = 6; 59) | 46.59*** (df = 1; 73) | 39.35*** (df = 5; 61) |

* p<0.05, **p<0.01,***p<0.001

*Table 4.* Results of univariable and multivariable regression models for female life expectancy at birth and male life expectancy at birth. Numbers in brackets are standard errors.

GSNI2 – Gender Social Norms Index 2; CVD – Cardiovascular disease; MMR – Maternal mortality ratio

For peer review only

## Discussion

We found a statistically significant relationship between higher country levels of biased gender social norms as measured by the Gender Social Norms Index and higher CVD mortality rates and lower life expectancy for both females and males. These results remained statistically significant after adjusting for country-level economic, education, and healthcare status measures. There was no statistically significant relationship demonstrated between the Gender Social Norms Index and female to male CVD mortality ratios within the adjusted model.

Despite a considerable body of research on the relationship between gender inequalities and health, there has been little to no previous quantitative investigation into how gender social norms may link to CVD and life expectancy. Kim *et al* have linked greater gender inequality as measured by the Gender Inequality Index to higher female to male stroke mortality ratios


For peer review only - http://bmjopen.bmj.com/site/about/guidelines.xhtml

and lower life expectancy, with use of similar ecological study designs[38-41]. However, some of these analyses did not control for confounding factors leaving results open to significant bias. The choice of covariates used in our study were designed to control for likely confounders within the realms of economics, education and healthcare provision at the country level, all known to have relationships with population health[29-31].

Additionally, previous research in the area of gender inequality and health has made use of the Gender Inequality Index as the primary explanatory variable[38-41]. However, linking this particular index to health outcomes may be confounded as the Gender Inequality Index includes maternal mortality ratio, which is also related to health outcomes. The use of the Gender Social Norms Index within our study avoids this limitation.

O'Neil *et al.* have proposed pathways through which gender influences norms, roles, attitudes and knowledge to affect exposure to psychosocial stressors and health behaviours which influence risk of CVD. For example, there is evidence that boys are socialised to take up more physical activity compared with girls, and adolescent girls are more likely to use smoking as a weight loss tool due to social pressures[16]. Additionally, the authors argue that traditional gender roles at home and work may be associated with worse cardiovascular health, including the expectations for men to be the sole breadwinner and women to take on care giving responsibilities[16]. The psychological impact of sexual abuse and gender discrimination has also been identified as a likely risk factor for CVD[16]. The results of our study support this theoretical framework with the suggestion at a country level of a significant association between gender social norms and population-level CVD outcomes and life expectancy.

For peer review only

*Strengths and limitations*

The strengths of this study include the robustness of the underlying data used, its global coverage, and novel use of the Gender Social Norms Index. Additionally, important potentially confounding factors have been adjusted for within the multivariable models. Sensitivity analysis was conducted demonstrating that all results were consistent across differing data time points apart from the male life expectancy outcome.

There are, however, important limitations to consider when interpreting the results of this analysis. Gender social norms are a complex social phenomenon and therefore challenging to measure and quantify, and the underlying data used to calculate the GSNI will likely be affected by selection bias, non-response bias and social desirability bias. Additionally, GSNI values were not available for all global countries.

The GSNI results included in this study were collected over a long period of time (2005-2014) and therefore there may be lack of standardisation between the data collected at different time points. It is hoped that including the GSNI collection period within the multivariable model will adjust for any systematic differences. However, whilst the GSNI values were not found to be statistically significantly different over time between the 2 waves of data collection, only 31 countries had data available for both time points. It may be the case that a country which had GSNI data collected in 2005 may have significantly changed norms in 2014, which is the time point used for the other data points within the main analysis. The sensitivity analysis ran the model with data from 2017, the results of which did not change in direction or significance apart from the male life expectancy outcome. However, it is noted that if GSNI data was collected in 2005 that remains over a decade in which gender social norms may have changed.

Ecological studies are utilised to measure population level variables and to influence policy at a regional and national scale[42]. The recognition of gender social norms as pervasive in the environment and influential beyond the individual was deemed an appropriate design for this


For peer review only - http://bmjopen.bmj.com/site/about/guidelines.xhtml

research question. However, it is well recognised that associations at the ecological level may not be seen at an individual level[43]. It could be argued that understanding the relationship between individually held gender social norms and individual health is an important but different question. However, the results of this research must be interpreted with caution in this regard.

Finally, the variables used as covariates with an unclear causal pathway may have introduced bias into the models. It is noted that the models demonstrate potentially unexpected covariate results including higher mean years of schooling and higher numbers of physicians per population associated with higher CVD mortality. This may be the result of countries with improving levels of such determinants experiencing increased burden from non-communicable diseases like CVD due to increased survival from other causes. As with any ecological study, it is essential to recognise this design cannot lead to any firm conclusions about a causal relationship between variables. However, our results suggest a strong positive and statistically significant association between gender social norms and CVD mortality that merits further exploration.

*Implications*

The results of this research imply there is an association between country level gender social norms and CVD mortality and life expectancy for males and females. The lack of association between gender social norms and female to male CVD mortality ratio suggests that gender social norms may affect both male and female CVD mortality rates equally. Based on the modelling results, a 10% improvement in a country's Gender Social Norms Index score could be associated with a 50 per 100 000 reduction in CVD mortality rates for both males and

For peer review only

females. Additionally, the total variation observed between countries in Gender Social Norm Index value could account for 6.3 years of life expectancy difference in females and 4.5 years in males. This supports the hypothesis that gender social norms are a social determinant of CVD and overall health more broadly. It is likely that there are additional pathways at play affecting the relationship between gender social norms and life expectancy besides its association with CVD mortality and this warrants further investigation. Theoretical explanations exist to explain the way gender and gender social norms can influence pathways to CVD outcomes including in health behaviours, healthcare and wider determinants of health[16]. However, an alternative explanation for the results could be that unmeasured societal factors that encourage more egalitarian gender social norms beyond better healthcare and country wealth, are also related to lower CVD mortality rates and longer life expectancy.

Biased gender social norms have been mooted as a potential reason the Sustainable Development Goals are not on target[2]. There is awareness of the importance of greater gender equality on the global stage however more must be done to reduce bias against minoritised genders[44]. Therefore, gender should be a prominent issue within the global health development agenda. The results of this study indicate that those working in this field should be open-minded to how gender inequality may be influencing a wide range of health problems for all genders. Particular consideration should be made for non-communicable diseases like CVD, as these are a growing problem for healthcare services and governments[45]. Campaigns such as the American Heart Association's Go Red for Women have recognised the importance of promoting heart health in women, raising awareness of symptoms, and encouraging more research to include women in clinical trials[46]. However, rather than focus solely on CVD in women and the healthcare biases that exist, such as the



For peer review only

For peer review only - http://bmjopen.bmj.com/site/about/guidelines.xhtml

lack of recognition of CVD symptoms in women and lack of applicable research[47], the findings of this study suggest the topic requires wider consideration. Not only around how gender social norms might influence broader CVD risk factors in women outside of the healthcare and research remit[48], but also how they influence people of all genders[49].

If another reason to promote and invest in gender equality is to reduce the burden of CVD, this could strengthen the argument for greater global investment in gender equity. Therefore, this research agenda could influence policy decision makers to consider the possible wider benefits of gender equality. It has been demonstrated that gender social norms can be influenced and changed by policy action, for example fair maternity and parental leave policies and ensuring education is free to all has been shown to change norms and improve health measures[5]. Additionally, programmes that aim to change gender social norms through multi-level, multi-sectoral and community empowerment have been evaluated to be effective[5]. Therefore, this research should be added to the ever-growing body of evidence that improving biased gender social norms and gender equality can contribute to better population health and should be a priority for policy decision makers as set out within the Sustainable Development Goals[1].

The relationships between gender social norms and CVD outcomes and life expectancy should be explored further with use of alternative measures of gender social norms, analysis over time and explore options for mediation analysis on the individual-level. Country case studies could also be explored for those with extreme values of the Gender Social Norms Index. The findings of this research suggest that it would be justified to investigate biased gender social norms as a phenomenon that may impact negatively on the entire population's health, in wide ranging ways, particularly in the area of non-communicable diseases.



For peer review only - http://bmjopen.bmj.com/site/about/guidelines.xhtml

For peer review only


For peer review only - http://bmjopen.bmj.com/site/about/guidelines.xhtml

***Glossary Box:***

***Sex -*** Biologically determined characteristics (genetics, hormones, anatomy) that lead to categorisation of individuals, typically into male, female and intersex.

***Gender -*** Considered a social construct, sociologically determined understandings and behaviours interacting with but separate from sex. Influenced by societal norms and changes over time.

***Social norms -*** The unwritten societal expectations influencing how we think and behave in a social situation, tend to be considered on an individual level i.e. how individuals react to what societal expectations exist.

***Gender norms -*** Engrained understandings of how individuals should act based on their gender, learnt through institutions such as family, school, workplace, religion, media and other social institutions. Conceptualised as a force external to individuals, upheld by institutions. Contributes to the perpetuation of the gender system which on the whole tends to favour males and masculinity.

view only



For peer review only - http://bmjopen.bmj.com/site/about/guidelines.xhtml

**Contributors**:

IL, AH and MOF conceived and designed the study. IL acquired and managed the data and completed all statistical analysis, which was reviewed by AH and MOF. IL wrote the first draft and is the guarantor for this study. SK and ML contributed to the critical discussion of the results and manuscript drafting. The corresponding author attests that all listed authors meet authorship criteria and that no others meeting the criteria have been omitted.

**Copyright:**

The Corresponding Author has the right to grant on behalf of all authors and does grant on behalf of all authors, a worldwide licence to the Publishers and its licensees in perpetuity, in all forms, formats and media (whether known now or created in the future), to i) publish, reproduce, distribute, display and store the Contribution, ii) translate the Contribution into other languages, create adaptations, reprints, include within collections and create summaries, extracts and/or, abstracts of the Contribution, iii) create any other derivative work(s) based on the Contribution, iv) to exploit all subsidiary rights in the Contribution, v) the inclusion of electronic links from the Contribution to third party material where-ever it may be located; and, vi) licence any third party to do any or all of the above.

**Funding:**

IL is an Academic Clinical Fellow funded by the UK National Institute for Health and Care Research in conjunction with Lancaster University. They are employed by St Helens and Knowsley NHS Foundation Trust.



For peer review only - http://bmjopen.bmj.com/site/about/guidelines.xhtml

AH is funded by a departmental studentship at the University of Liverpool and supported by the UK National Institute for Health and Care Research School for Public Health (Grant Reference Number PD-SPH-2015-10025).

SK receives funding from the National Heart, Lung and Blood Institute and the American Heart Association.

These funders had no role in the study design, analysis or presentation of results. The views expressed are those of the authors and not necessarily those of the National Institute for Health and Care Research, the NHS, the National Heart, Lung and Blood Institute or the American Heart Association.

**Competing interests:**

All authors have completed the ICMJE uniform disclosure form at www.icmje.org/coi_disclosure.pdf and declare: no support from any organisation for the submitted work; no financial relationships with any organisations that might have an interest in the submitted work in the previous three years; no other relationships or activities that could appear to have influenced the submitted work.

**Ethical approval:** No ethics committee approval required.

**Transparency statement**: The manuscript's lead author and guarantor (IL) affirms that the manuscript is an honest, accurate, and transparent account of the study being reported; no important aspects of the study have been omitted and any discrepancies from the study as originally planned have been explained.

For peer review only

**Data sharing:** The data used in this analysis are openly available from the Institute for Health Metrics and Evaluation, The World Bank, The Global Health Observatory, the United Nations Development Programme and Our World in Data. Code used for analysis is available via GitHub here.

**Liscence statement:** I, the Submitting Author has the right to grant and does grant on behalf of all authors of the Work (as defined in the author licence), an exclusive licence and/or a non-exclusive licence for contributions from authors who are: i) UK Crown employees; ii) where BMJ has agreed a CC-BY licence shall apply, and/or iii) in accordance with the terms applicable for US Federal Government officers or employees acting as part of their official duties; on a worldwide, perpetual, irrevocable, royalty-free basis to BMJ Publishing Group Ltd ("BMJ") its licensees.

The Submitting Author accepts and understands that any supply made under these terms is made by BMJ to the Submitting Author unless you are acting as an employee on behalf of your employer or a postgraduate student of an affiliated institution which is paying any applicable article publishing charge ("APC") for Open Access articles. Where the Submitting Author wishes to make the Work available on an Open Access basis (and intends to pay the relevant APC), the terms of reuse of such Open Access shall be governed by a Creative Commons licence – details of these licences and which licence will apply to this Work are set out in our licence referred to above.



For peer review only - http://bmjopen.bmj.com/site/about/guidelines.xhtml

## References

1. United Nations. The Sustainable Development Goals Report 2020. 2020. United Nations. https://unstats.un.org/sdgs/report/2020/The-Sustainable-Development-Goals-Report-2020.pdf

2. Clark J and Horton R. A coming of age for gender in global health. The Lancet 2019; 393: 2367-2369.

3. Hay K, McDougal L, Percival V, et al. Disrupting gender norms in health systems: making the case for change. The Lancet 2019; 393: 2535-2549.

4. Heise L, Greene ME, Opper N, et al. Gender inequality and restrictive gender norms: framing the challenges to health. The Lancet 2019; 393: 2440-2454.

5. Heymann J, Levy JK, Bose B, et al. Improving health with programmatic, legal, and policy approaches to reduce gender inequality and change restrictive gender norms. The Lancet 2019; 393: 2522-2534.

6. Weber AM, Cislaghi B, Meausoone V, et al. Gender norms and health: insights from global survey data. The Lancet 2019; 393: 2455-2468.

7. Mejía-Guevara I, Cislaghi B, Weber A, et al. Association of collective attitudes and contraceptive practice in nine sub-Saharan African countries. Journal of global health 2020; 10.

8. Shakya HB, Darmstadt GL, Barker KM, et al. Social normative and social network factors associated with adolescent pregnancy: a cross-sectional study of 176 villages in rural Honduras. Journal of global health 2020; 10: 010706-010706.

9. Stark L, Seff I, Weber AM, et al. Perpetration of intimate partner violence and mental health outcomes: sex- and gender-disaggregated associations among adolescents and young adults in Nigeria. Journal of global health 2020; 10: 010708-010708.

10. Omidakhsh N and Heymann J. Improved child marriage laws and its association with changing attitudes and experiences of intimate partner violence: a comparative multi-national study. Journal of global health 2020; 10: 010707-010707.

11. Bonita R and Beaglehole R. Women and NCDs: overcoming the neglect. Global health action 2014; 7: 23742. 2014/05/09.

12. Homan P. Political gender inequality and infant mortality in the United States, 1990–2012. Social Science & Medicine 2017; 182: 127-135.

13. Heaton TB. Are Improvements in Child Health Due to Increasing Status of Women in Developing Nations? Biodemography and Social Biology 2015; 61: 252-265.

14. Stark L, Seff I, Weber A, et al. Applying a gender lens to global health and well-being: Framing a Journal of Global Health special collection. Journal of global health 2020; 10: 010103. 2020/04/08.



For peer review only - http://bmjopen.bmj.com/site/about/guidelines.xhtml

15. Vos T, Lim SS, Abbafati C, et al. Global burden of 369 diseases and injuries in 204 countries and territories, 1990–2019: a systematic analysis for the Global Burden of Disease Study 2019. The Lancet 2020; 396: 1204-1222.

16. O'Neil A, Scovelle AJ, Milner AJ, et al. Gender/Sex as a Social Determinant of Cardiovascular Risk. Circulation 2018; 137: 854-864.

17. World Health Organisation. 2021. Gender and Health, https://www.who.int/health-topics/gender#tab=tab_1 (accessed 09/07/2021).

18. Kim ES and Menon V. Status of women in cardiovascular clinical trials. Arteriosclerosis, thrombosis, and vascular biology 2009; 29: 279-283.

19. Cislaghi B and Heise L. Gender norms and social norms: differences, similarities and why they matter in prevention science. Sociology of health & illness 2020; 42: 407-422. https://doi.org/10.1111/1467-9566.13008.

20. Walli-Attaei M, Joseph P, Rosengren A, et al. Variations between women and men in risk factors, treatments, cardiovascular disease incidence, and death in 27 high-income, middle-income, and low-income countries (PURE): a prospective cohort study. The Lancet 2020; 396: 97-109.

21. Liakos M and Parikh PB. Gender Disparities in Presentation, Management, and Outcomes of Acute Myocardial Infarction. Current Cardiology Reports 2018; 20: 64.

22. Manandhar M, Hawkes S, Buse K, et al. Gender, health and the 2030 agenda for sustainable development. Bulletin of the World Health Organization 2018; 96: 644-653. 2018/06/12.

23. Woodward M. Cardiovascular Disease and the Female Disadvantage. International Journal of Environmental Research and Public Health 2019; 16 2019/04/04.

24. Wang H, Abbas KM, Abbasifard M, et al. Global age-sex-specific fertility, mortality, healthy life expectancy (HALE), and population estimates in 204 countries and territories, 1950–2019: a comprehensive demographic analysis for the Global Burden of Disease Study 2019. The Lancet 2020; 396: 1160-1203.

25. The Global Health Observatory. 2021. Life expectancy at birth (years), https://www.who.int/data/gho/data/indicators/indicator-details/GHO/life-expectancy-at-birth-(years) (accessed 14/07/2021).

26. Institute for Health Metrics and Evaluation. 2021. GBD Compare, https://vizhub.healthdata.org/gbd-compare/le (accessed 07/07/2021).

27. Institute for Future Studies. 2021. World Values Survey, https://www.iffs.se/en/world-values-survey/ (accessed 14/07/2021).

28. United Nations Development Programme. Frequenty Asked Questions - Gender Social Norms Index (GSNI). 2020. http://hdr.undp.org/sites/default/files/frequently_asked_questions_gsni.pdf

For peer review only

29. Swift R. The relationship between health and GDP in OECD countries in the very long run. Health Economics 2011; 20: 306-322.

30. Cutler DM and Lleras-Muney A. Education and health: insights from international comparisons. 2012.

31. Basu S, Berkowitz SA, Phillips RL, et al. Association of Primary Care Physician Supply With Population Mortality in the United States, 2005-2015. JAMA Internal Medicine 2019; 179: 506-514.

32. The World Bank. 2021. GDP per capita (current US$), https://data.worldbank.org/indicator/NY.GDP.PCAP.CD (accessed 01/06/2021).

33. The World Bank. 2021. Physicians (per 1,000 people), https://data.worldbank.org/indicator/SH.MED.PHYS.ZS (accessed 01/06/2021).

34. Roser M and Ortiz-Ospina E. 2016. Global Education, https://ourworldindata.org/global-education (accessed 03/06/2021).

35. The World Bank. 2021. Maternal mortality ratio (modeled estimate, per 100,000 live births), https://data.worldbank.org/indicator/SH.STA.MMRT (accessed 01/06/2021).

36. Bruce N, Pope D and Stanistreet D. Quantitative methods for health research. Wiley Online Library, 2017. 1118665376.

37. Leach LF and Henson RK. The use and impact of adjusted R2 effects in published regression research. Multiple Linear Regression Viewpoints 2007; 33: 1-11.

38. Kim JI and Kim G. Labor force participation and secondary education of gender inequality index (GII) associated with healthy life expectancy (HLE) at birth. International Journal for Equity in Health 2014; 13: 106.

39. Kim JI and Kim G. Socio-ecological perspective of older age life expectancy: income, gender inequality, and financial crisis in Europe. Globalization & Health 2017; 13: 1-8.

40. Kim JI and Kim G. Country-level socioeconomic indicators associated with survival probability of becoming a centenarian among older european adults: gender inequality, male labour force participation and proportions of women in parliaments. Journal of biosocial science 2017; 49: 239-250.

41. Kim YD, Jung YH, Caso V, et al. Countries with women inequalities have higher stroke mortality. International journal of stroke : official journal of the International Stroke Society 2017; 12: 869-874.

42. Adeyinka DA, Evans MR, Ozigbu CE, et al. Understanding the Influence of Socioeconomic Environment on Paediatric Antiretroviral Treatment Coverage: Towards Closing Treatment Gaps in Sub-Saharan Africa. Central European journal of public health 2017; 25: 55-63.

43. Sedgwick P. Ecological studies: advantages and disadvantages. BMJ 2014; 348: g2979.

For peer review only

44. Darmstadt GL, Heise L, Gupta GR, et al. Why now for a Series on gender equality, norms, and health? The Lancet 2019; 393: 2374-2377.

45. Bennett JE, Kontis V, Mathers CD, et al. NCD Countdown 2030: pathways to achieving Sustainable Development Goal target 3.4. The Lancet 2020; 396: 918-934.

46. American Heart Association. 2021. Go Red for Women, https://www.goredforwomen.org/en (accessed 06/08/2021).

47. Aggarwal NR, Patel HN, Mehta LS, et al. Sex Differences in Ischemic Heart Disease: Advances, Obstacles, and Next Steps. Circulation: Cardiovascular Quality and Outcomes 2018; 11: e004437. 2018/02/17.

48. Lindley Kathryn J, Aggarwal Niti R, Briller Joan E, et al. Socioeconomic Determinants of Health and Cardiovascular Outcomes in Women. Journal of the American College of Cardiology 2021; 78: 1919-1929.

49. Emslie C and Hunt K. Men, Masculinities and Heart Disease. Current Sociology - CURR SOCIOL 2009; 57: 155-191.

For peer review only



For peer review only - http://bmjopen.bmj.com/site/about/guidelines.xhtml

## Figure legend

**Figure 1.** Scatter plots of GSNI2 against female, male and population age standardised CVD mortality rates. (GSNI2 – Gender Social Norms Index 2; CVD – Cardiovascular disease)

**Figure 2.** Scatter plots of GSNI2 against female and male life expectancy. (GSNI2 – Gender Social Norms Index 2)

For peer review only

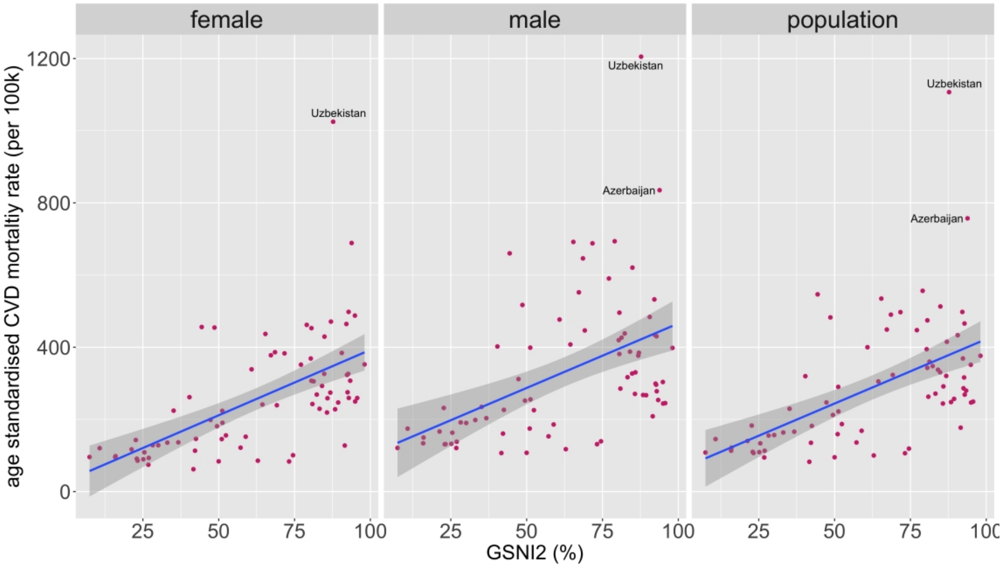


Figure 1. Scatter plots of GSNI2 against female, male and population age standardised CVD mortality rates. (GSNI2 – Gender Social Norms Index 2; CVD – Cardiovascular disease)

1458x833mm (72 x 72 DPI)

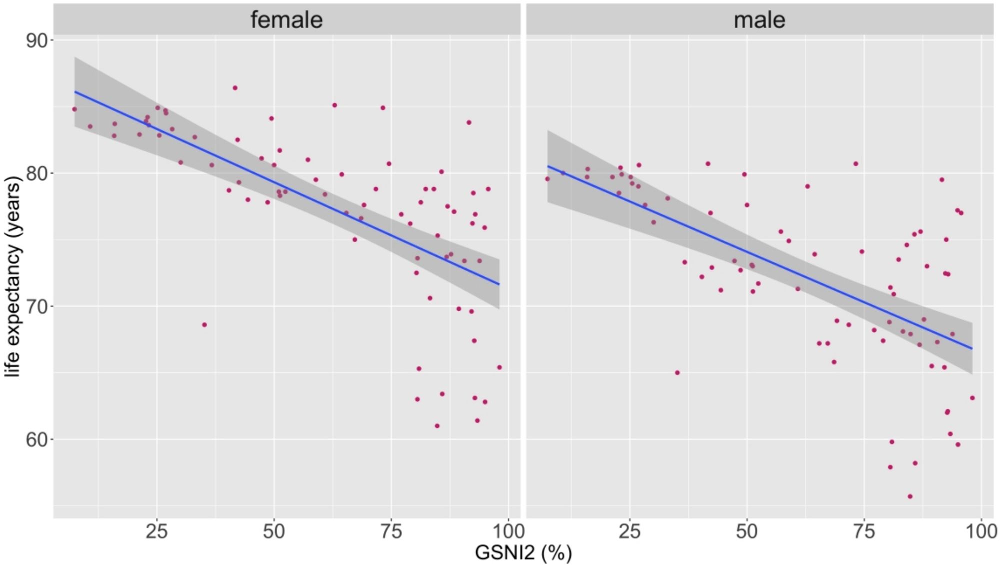


Figure 2. Scatter plots of GSNI2 against female and male life expectancy. (GSNI2 – Gender Social Norms Index 2)

1458x833mm (72 x 72 DPI)

Supplemental Material:

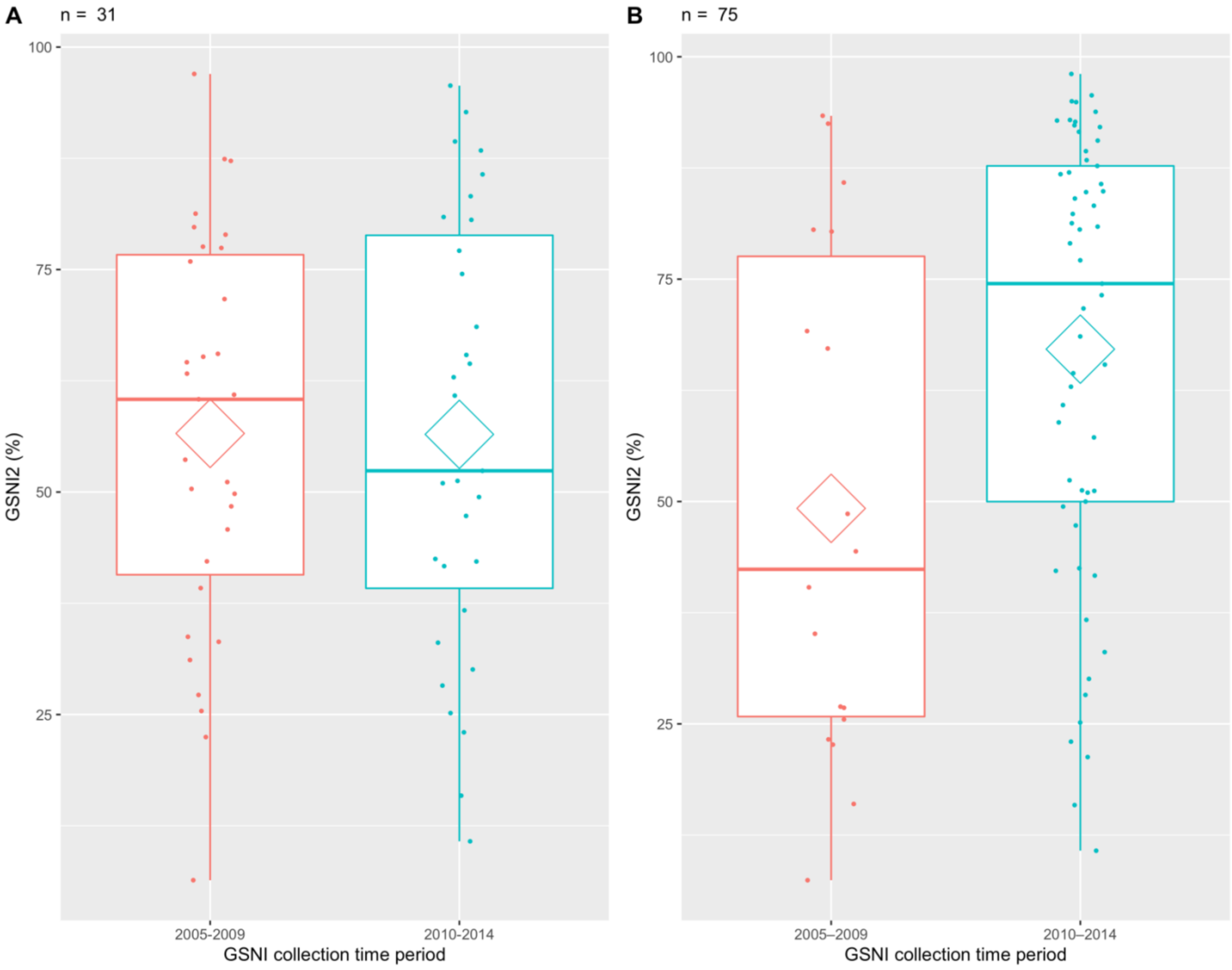


**Figure S1. A.** Box plots comparing GSNI2 values between countries that had values for both 2005-2009 collection period and 2010-2014 collection period. **B.** Box plots splitting the GSNI2 values included in the analysis by period of collection.

**Table S1.** List of countries used within univariable analyses n=75

| | | |
|---|---|---|
| Algeria | India | Poland |
| Andorra | Indonesia | Qatar |
| Argentina | Iran, Islamic Republic of | Romania |
| Armenia | Iraq | Russian Federation |
| Australia | Japan | Rwanda |
| Azerbaijan | Jordan | Serbia |
| Belarus | Kazakhstan | Singapore |
| Brazil | Korea (Republic of) | Slovenia |
| Bulgaria | Kuwait | South Africa |
| Burkina Faso | Kyrgyzstan | Spain |
| Canada | Lebanon | Sweden |
| Chile | Libya | Switzerland |
| China | Malaysia | Thailand |
| Colombia | Mali | Trinidad and Tobago |
| Cyprus | Mexico | Tunisia |
| Ecuador | Moldova, Republic of | Turkey |
| Estonia | Morocco | Ukraine |
| Ethiopia | Netherlands | United Kingdom |
| Finland | New Zealand | United States |
| France | Nigeria | Uruguay |
| Georgia | Norway | Uzbekistan |
| Germany | Pakistan | Viet Nam |
| Ghana | Palestine, State of | Yemen |
| Haiti | Peru | Zambia |
| Hungary | Philippines | Zimbabwe |

For peer review only

**Table S2.** List of countries used within multivariable analyses (Andorra not included in female life expectancy outcome as maternal mortality ratio not available; Qatar not included in CVD ratio outcome as identified as an influential outlier)
n=67

| | | |
|---|---|---|
| Algeria | Ghana | Philippines |
| Andorra | Haiti | Poland |
| Argentina | Hungary | Qatar |
| Armenia | India | Romania |
| Australia | Indonesia | Rwanda |
| Azerbaijan | Iraq | Serbia |
| Belarus | Japan | Singapore |
| Brazil | Jordan | Slovenia |
| Bulgaria | Kazakhstan | South Africa |
| Burkina Faso | Kuwait | Spain |
| Canada | Lebanon | Sweden |
| Chile | Libya | Switzerland |
| China | Malaysia | Thailand |
| Colombia | Mali | Trinidad and Tobago |
| Cyprus | Mexico | Tunisia |
| Ecuador | Morocco | Turkey |
| Estonia | Netherlands | Ukraine |
| Ethiopia | New Zealand | United Kingdom |
| Finland | Nigeria | United States |
| France | Norway | Uruguay |
| Georgia | Pakistan | Uzbekistan |
| Germany | Peru | Zambia |
| | | Zimbabwe |

For peer review only

| | *Dependent variable:* | | | |
|---|---|---|---|---|
| | female CVD mortality 2017 | | male CVD mortality 2017 | |
| | (1) | (2) | (3) | (4) |
| constant | 27.10 (37.18) | -118.15 (91.70) | 99.23 (50.56) | -181.55 (105.32) |
| GSNI2 | 3.57*** (0.55) | 4.27*** (0.85) | 3.59*** (0.74) | 4.78*** (0.98) |
| physicians per 1000 | | 8.39 (15.71) | | 39.43* (18.04) |
| mean years of schooling | | 20.53* (8.80) | | 29.30** (10.10) |
| GDP per capita | | -0.002* (0.001) | | -0.004*** (0.001) |
| GSNI data collection period 2010-2014 | | -90.81* (38.64) | | -115.70* (44.37) |
| $R^2$ | 0.37 | 0.49 | 0.24 | 0.55 |
| Adjusted $R^2$ | 0.36 | 0.45 | 0.23 | 0.51 |
| F Statistic | 42.72*** (df = 1; 73) | 11.80*** (df = 5; 61) | 23.38*** (df = 1; 73) | 14.80*** (df = 5; 61) |

* p<0.05, **p<0.01,***p<0.001

**Table S3.** Results of the sensitivity analysis univariable and multivariable regression models for the female and male CVD mortality outcomes. Numbers in brackets are standard errors. GSNI2 – Gender Social Norms Index 2; CVD – Cardiovascular disease

For peer review only

| | *Dependent variable:* | | | |
|---|---|---|---|---|
| | CVD mortality 2017 | | female to male CVD mortality ratio 2017 | |
| | (1) | (2) | (3) | (4) |
| constant | 60.45 (41.48) | -132.22 (93.98) | $0.58^{***}$ (0.05) | $0.92^{***}$ (0.10) |
| GSNI2 | $3.54^{***}$ (0.61) | $4.36^{***}$ (0.87) | $0.003^{***}$ (0.001) | 0.001 (0.001) |
| physicians per 1000 | | 22.02 (16.10) | | $-0.04^{*}$ (0.02) |
| mean years of schooling | | $23.92^{*}$ (9.01) | | -0.01 (0.01) |
| GDP per capita | | $-0.003^{**}$ (0.001) | | -0.0000 (0.0000) |
| GSNI data collection period 2010-2014 | | $-101.63^{*}$ (39.59) | | -0.03 (0.04) |
| $R^2$ | 0.32 | 0.53 | 0.22 | 0.44 |
| Adjusted $R^2$ | 0.31 | 0.49 | 0.21 | 0.39 |
| F Statistic | $33.75^{***}$ (df = 1; 73) | $13.60^{***}$ (df = 5; 61) | $20.31^{***}$ (df = 1; 73) | $9.38^{***}$ (df = 5; 60) |

* p<0.05, **p<0.01,***p<0.001

**Table S4.** Results of the sensitivity analysis univariable and multivariable regression models for CVD mortality and female to male CVD mortaliy ratio outcomes Numbers in brackets are standard errors.
GSNI2 – Gender Social Norms Index 2; CVD – Cardiovascular disease

For peer review only

| | *Dependent variable:* | | | |
|---|---|---|---|---|
| | female life expectancy 2019 | | male life expectancy 2019 | |
| | (1) | (2) | (3) | (4) |
| constant | 87.73*** (1.36) | 60.75*** (4.23) | 82.27*** (1.42) | 38.63*** (5.06) |
| GSNI | -0.15*** (0.02) | -0.04* (0.02) | -0.15*** (0.02) | -0.02 (0.02) |
| log (physicians per 1000) | | 0.92** (0.28) | | 0.81* (0.36) |
| mean years of schooling | | -0.29 (0.17) | | -0.47 (0.23) |
| log (GDP per capita) | | 2.46*** (0.43) | | 4.28*** (0.56) |
| GSNI data collection period 2010-2014 | | 1.32 (0.78) | | 1.12 (1.06) |
| MMR | | -0.01*** (0.003) | | |
| $R^2$ | 0.45 | 0.88 | 0.40 | 0.77 |
| Adjusted $R^2$ | 0.44 | 0.87 | 0.39 | 0.75 |
| F Statistic | 58.99*** (df = 1; 73) | 70.88*** (df = 6; 59) | 48.54*** (df = 1; 73) | 40.03*** (df = 5; 61) |

* p<0.05, **p<0.01,***p<0.001

**Table S5.** Results of the sensitivity analysis univariable and multivariable regression models for female and male life expectancy at birth. Numbers in brackets are standard errors. GSNI2 – Gender Social Norms Index 2; CVD – Cardiovascular disease; MMR – Maternal mortality ratio

For peer review only

STROBE Statement—checklist of items that should be included in reports of observational studies

| | Item No | Recommendation | Page No |
|---|---|---|---|
| **Title and abstract** | 1 | (*a*) Indicate the study's design with a commonly used term in the title or the abstract | 1 |
| | | (*b*) Provide in the abstract an informative and balanced summary of what was done and what was found | 4 |
| **Introduction** | | | |
| Background/rationale | 2 | Explain the scientific background and rationale for the investigation being reported | 5 |
| Objectives | 3 | State specific objectives, including any prespecified hypotheses | 5 |
| **Methods** | | | |
| Study design | 4 | Present key elements of study design early in the paper | 6 |
| Setting | 5 | Describe the setting, locations, and relevant dates, including periods of recruitment, exposure, follow-up, and data collection | 6 |
| Participants | 6 | (*a*) *Cohort study*—Give the eligibility criteria, and the sources and methods of selection of participants. Describe methods of follow-up<br>*Case-control study*—Give the eligibility criteria, and the sources and methods of case ascertainment and control selection. Give the rationale for the choice of cases and controls<br>*Cross-sectional study*—Give the eligibility criteria, and the sources and methods of selection of participants | 6 |
| | | (*b*) *Cohort study*—For matched studies, give matching criteria and number of exposed and unexposed<br>*Case-control study*—For matched studies, give matching criteria and the number of controls per case | NA |
| Variables | 7 | Clearly define all outcomes, exposures, predictors, potential confounders, and effect modifiers. Give diagnostic criteria, if applicable | 6-7 |
| Data sources/ measurement | 8* | For each variable of interest, give sources of data and details of methods of assessment (measurement). Describe comparability of assessment methods if there is more than one group | 6-7 |
| Bias | 9 | Describe any efforts to address potential sources of bias | 6-8 |
| Study size | 10 | Explain how the study size was arrived at | NA |
| Quantitative variables | 11 | Explain how quantitative variables were handled in the analyses. If applicable, describe which groupings were chosen and why | 7-8 |
| Statistical methods | 12 | (*a*) Describe all statistical methods, including those used to control for confounding | 7-8 |
| | | (*b*) Describe any methods used to examine subgroups and interactions | NA |
| | | (*c*) Explain how missing data were addressed | 7-8 |
| | | (*d*) *Cohort study*—If applicable, explain how loss to follow-up was addressed<br>*Case-control study*—If applicable, explain how matching of cases and controls was addressed<br>*Cross-sectional study*—If applicable, describe analytical methods taking account of sampling strategy | NA |
| | | (*e*) Describe any sensitivity analyses | 7-8 |

Continued on next page



For peer review only - http://bmjopen.bmj.com/site/about/guidelines.xhtml

| Results | | | |
|---|---|---|---|
| Participants | 13* | (a) Report numbers of individuals at each stage of study—eg numbers potentially eligible, examined for eligibility, confirmed eligible, included in the study, completing follow-up, and analysed | NA |
| | | (b) Give reasons for non-participation at each stage | NA |
| | | (c) Consider use of a flow diagram | NA |
| Descriptive data | 14* | (a) Give characteristics of study participants (eg demographic, clinical, social) and information on exposures and potential confounders | 15-16 |
| | | (b) Indicate number of participants with missing data for each variable of interest | 15-16 |
| | | (c) *Cohort study*—Summarise follow-up time (eg, average and total amount) | NA |
| Outcome data | 15* | *Cohort study*—Report numbers of outcome events or summary measures over time | NA |
| | | *Case-control study*—Report numbers in each exposure category, or summary measures of exposure | NA |
| | | *Cross-sectional study*—Report numbers of outcome events or summary measures | 15-16 |
| Main results | 16 | (*a*) Give unadjusted estimates and, if applicable, confounder-adjusted estimates and their precision (eg, 95% confidence interval). Make clear which confounders were adjusted for and why they were included | 18-20 |
| | | (*b*) Report category boundaries when continuous variables were categorized | NA |
| | | (*c*) If relevant, consider translating estimates of relative risk into absolute risk for a meaningful time period | NA |
| Other analyses | 17 | Report other analyses done—eg analyses of subgroups and interactions, and sensitivity analyses | 10 |
| **Discussion** | | | |
| Key results | 18 | Summarise key results with reference to study objectives | 10 |
| Limitations | 19 | Discuss limitations of the study, taking into account sources of potential bias or imprecision. Discuss both direction and magnitude of any potential bias | 11-12 |
| Interpretation | 20 | Give a cautious overall interpretation of results considering objectives, limitations, multiplicity of analyses, results from similar studies, and other relevant evidence | 12-14 |
| Generalisability | 21 | Discuss the generalisability (external validity) of the study results | 11 |
| **Other information** | | | |
| Funding | 22 | Give the source of funding and the role of the funders for the present study and, if applicable, for the original study on which the present article is based | 23 |

*Give information separately for cases and controls in case-control studies and, if applicable, for exposed and unexposed groups in cohort and cross-sectional studies.

**Note:** An Explanation and Elaboration article discusses each checklist item and gives methodological background and published examples of transparent reporting. The STROBE checklist is best used in conjunction with this article (freely available on the Web sites of PLoS Medicine at http://www.plosmedicine.org/, Annals of Internal Medicine at http://www.annals.org/, and Epidemiology at http://www.epidem.com/). Information on the STROBE Initiative is available at www.strobe-statement.org.


For peer review only - http://bmjopen.bmj.com/site/about/guidelines.xhtml

BMJ Open

# The association between gender social norms and cardiovascular disease mortality and life expectancy: an ecological study

| | |
|---|---|
| Journal: | *BMJ Open* |
| Manuscript ID | bmjopen-2022-065486.R1 |
| Article Type: | Original research |
| Date Submitted by the Author: | 19-Mar-2023 |
| Complete List of Authors: | Lyell, Iona; Lancaster University, Faculty of Health and Medicine<br>Khan, S; Northwestern University Feinberg School of Medicine<br>Limmer, Mark; Lancaster University, Faculty of Health and Medicine<br>O'Flaherty, M; University of Liverpool, Department of Public Health & Policy<br>Head, Anna; University of Liverpool, Department of Public Health & Policy |
| <b>Primary Subject Heading</b>: | Global health |
| Secondary Subject Heading: | Public health |
| Keywords: | Cardiac Epidemiology < CARDIOLOGY, PUBLIC HEALTH, EPIDEMIOLOGY |
| | |

SCHOLARONE™
Manuscripts

*I, the Submitting Author has the right to grant and does grant on behalf of all authors of the Work (as defined in the below author licence), an exclusive licence and/or a non-exclusive licence for contributions from authors who are: i) UK Crown employees; ii) where BMJ has agreed a CC-BY licence shall apply, and/or iii) in accordance with the terms applicable for US Federal Government officers or employees acting as part of their official duties; on a worldwide, perpetual, irrevocable, royalty-free basis to BMJ Publishing Group Ltd ("BMJ") its licensees and where the relevant Journal is co-owned by BMJ to the co-owners of the Journal, to publish the Work in this journal and any other BMJ products and to exploit all rights, as set out in our licence.*

*The Submitting Author accepts and understands that any supply made under these terms is made by BMJ to the Submitting Author unless you are acting as an employee on behalf of your employer or a postgraduate student of an affiliated institution which is paying any applicable article publishing charge ("APC") for Open Access articles. Where the Submitting Author wishes to make the Work available on an Open Access basis (and intends to pay the relevant APC), the terms of reuse of such Open Access shall be governed by a Creative Commons licence – details of these licences and which Creative Commons licence will apply to this Work are set out in our licence referred to above.*

*Other than as permitted in any relevant BMJ Author's Self Archiving Policies, I confirm this Work has not been accepted for publication elsewhere, is not being considered for publication elsewhere and does not duplicate material already published. I confirm all authors consent to publication of this Work and authorise the granting of this licence.*

For peer review only

**Title:**

The association between gender social norms and cardiovascular disease mortality and life expectancy: an ecological study

**Authors:**

Iona Lyell, Sadiya S. Khan, Mark Limmer, Martin O'Flaherty, Anna Head

**Address for Correspondence:**

Anna Head

Room 3.13, Whelan Building

University of Liverpool, Liverpool, L69 3DT

United Kingdom

Tel: +44 (0) 151 795 2664

Email: anna.head@liverpool.ac.uk

**Institutions:**

Faculty of Health and Medicine, Lancaster University, Lancaster, UK

Iona Lyell

Academic Clinical Fellow in Public Health

Northwestern University, Chicago, USA

Sadiya S. Khan

Assistant Professor of Medicine (Cardiology) and Preventative Medicine (Epidemiology)

For peer review only

Faculty of Health and Medicine, Lancaster University, Lancaster, UK

Mark Limmer

Senior Lecturer

Department of Public Health, Policy and Systems, University of Liverpool, Liverpool, UK

Martin O'Flaherty

Professor in Epidemiology

Department of Public Health, Policy and Systems, University of Liverpool, Liverpool, UK

Anna Head

Doctoral Candidate

**Key words (MeSH):** epidemiology; global health; cardiovascular diseases; life expectancy; sexism

**Word count: 3526**



For peer review only - http://bmjopen.bmj.com/site/about/guidelines.xhtml

# Abstract

**Objective** Examine the association between country-level gender social norms and 1) cardiovascular disease mortality rates; 2) female to male cardiovascular disease mortality ratios; and 3) life expectancy.

**Design** Ecological study with the country as the unit of analysis.

**Setting** Global, country-level data.

**Participants** Global population of countries with data available on gender social norms as measured by the Gender Social Norms Index (developed by the United Nations Development Programme).

**Main outcome measures** Country-level female and male age-standardised cardiovascular disease mortality rates, population age-standardised cardiovascular disease mortality rates, female to male cardiovascular disease mortality ratios, female and male life expectancy at birth. Outcome measure data were retrieved from the WHO and the Institute for Health Metrics and Evaluation. Multivariable linear regression models were fitted to explore the relationship between gender social norms and the outcome variables.

**Results** Higher levels of biased gender social norms, as measured by the Gender Social Norms Index, were associated with higher female, male, and population cardiovascular disease mortality rates in the multivariable models ($\beta$ 4.86, 95% CIs 3.18 to 6.54; $\beta$ 5.28, 95% CIs 3.42 to 7.15; $\beta$ 4.89, 95% CIs 3.18 to 6.60), and lower female and male life expectancy ($\beta$ -0.07, 95% CIs -0.11 to -0.03; $\beta$ -0.05, 95% CIs -0.10 to -0.01). These results included adjustment within the models for potentially confounding country level factors



For peer review only - http://bmjopen.bmj.com/site/about/guidelines.xhtml

For peer review only

including GDP per capita, population mean years of schooling, physicians per 1000 population, year of Gender Social Norms Index data collection and maternal mortality ratio.

**Conclusions** Our analysis suggests that higher levels of biased gender social norms are associated with higher rates of population cardiovascular disease mortality and lower life expectancy for both sexes. Future research should explore this relationship further, to define its causal role and promote public health action.

For peer review only



For peer review only - http://bmjopen.bmj.com/site/about/guidelines.xhtml

Strengths and Limitations:

- The Gender Social Norms Index has been used as a quantified measure of gender social norms at country level for 75 countries
- The outcome measures of cardiovascular disease mortality and life expectancy were stratified by male and female
- Confounding variables were included within the analysis to take into account underlying differences between countries
- Data was not available to analyse the relationship between variables over time

For peer review only


For peer review only - http://bmjopen.bmj.com/site/about/guidelines.xhtml

## Introduction

The population health benefits of gender equity are well recognised within the global health research community and reflected in development priorities including the Sustainable Development Goals[1, 2]. However, research in the area of gender social norms and health has thus far been largely focused on issues classically considered as 'women's health' related (reproduction, child health and intimate partner violence[3-14]) and there is a lack of understanding in terms of the relationship with non-communicable diseases at a population level. It is cardiovascular disease (CVD) specifically that is the leading cause of death worldwide[15] and a significant cause of premature mortality globally for both women and men[15].

Gender and the norms associated with it are considered a construct of society, influenced by but separate from biological sex[16] *(see glossary box)*. A means through which power dynamics flow within societies, gender determines actions and reactions of individuals and communities and therefore acts as a social determinant of health[16, 17]. Gender social norms are the 'unspoken rules' influencing how individuals enact their gender and interact with others[6, 18]. Theoretical explanations exist to link gender and gender social norms to CVD outcomes including in healthcare, health behaviours and wider determinants of health[19]. These include a recognition of gender bias in research and treatment of CVD, an understanding that gender social norms influence patterns of CVD risk factors e.g. stress and smoking, and their impact on important social determinants of CVD including education and employment[17, 20, 21]. Therefore, whilst there are theoretical explanations for a connection between gender social


For peer review only - http://bmjopen.bmj.com/site/about/guidelines.xhtml

norms and CVD mortality at a population level[19], this has not yet been explored in an ecological framework with use of country level data.

It is theorised that increased levels of biased gender social norms may be associated with determinants and behaviours harmful to cardiovascular health on a population-level, and therefore an increased CVD burden and reduced life expectancy more broadly for both women and men[19, 22]. Therefore, this study aimed to explore the association between gender social norms, as measured by the Gender Social Norms Index and CVD mortality and life expectancy at the country level.

## Methods

This research made use of an ecological study design with the country as the unit of analysis to investigate the relationship between gender social norms and CVD mortality rates, female to male CVD mortality ratios and life expectancy.

### *Outcome variables*

The outcome variables were defined as; female, male and total age-standardised CVD mortality rate per 100 000 population, female to male CVD mortality ratio, female and male life expectancy at birth in years. The CVD outcome data was derived from the Global Burden of Disease (GBD) study 2019[15]. Age-standardised rates were calculated with use of the GBD world population age standard[15], calculated from the GBD 2019 population estimates[23]. CVD as cause of death in the GBD 2019 includes 11 disease categories: ischaemic heart disease, stroke, hypertensive heart disease, atrial fibrillation/flutter, rheumatic heart disease, non-


For peer review only - http://bmjopen.bmj.com/site/about/guidelines.xhtml

rheumatic valvular heart disease, cardiomyopathy and myocarditis, aortic aneurysm, peripheral artery disease, endocarditis and other cardiovascular and circulatory diseases[15]. Life expectancy at birth data was downloaded from the Global Health Observatory hosted by the WHO[24]. It is defined as 'the average number of years that a newborn could expect to live, if he or she were to pass through life exposed to the sex and age specific death rates prevailing at the time of his or her birth, for a specific year, in a given country, territory, or geographic area.'[24] Three countries (Andorra, UK, and Palestine) did not have data available from this source, instead this data was retrieved from the Institute for Health Metrics and Evaluation[25].

*Primary explanatory variable*

The Gender Social Norms Index (GSNI) was created by the United Nations Development Programme and available to download from http://hdr.undp.org/en/gsni. It is a novel, freely available, quantitative measure of gender social norms at the country level and derived from the World Values Survey[26]. The data was collected in two waves of the survey undertaken between 2005-2009 and 2010-2014. The GSNI is made up of four domains (political, educational, economic and physical integrity). The share of people with at least two gender biases (GSNI2) was chosen as the primary explanatory variable as it was presumed this would demonstrate a stronger relationship if there was one to be found compared with the share of people with one bias. Values are provided as a percentage for each country. The value for GSNI2 can be interpreted as an estimate of the prevalence in a country of moderate to intense gender bias[27]. 75 countries have an index value available which amounts to the representation of 81% of the global population[27].


For peer review only - http://bmjopen.bmj.com/site/about/guidelines.xhtml

*Covariates*

GDP per capita, physicians per 1000 population and mean years of schooling were included as additional country level covariates based on evidence of the association of these variables with population health[28-30]. Datasets for these variables were retrieved from The World Bank and Our World in Data[31-33]. Maternal mortality ratio was included as a covariate in the female life expectancy model and was retrieved from The World Bank[34]. It is possible that the wave of GSNI data collection may have influenced the results e.g. through passage of time or changes in methodology. Therefore, this variable was also included within the model.

*Choice of variables (year)*

Variables (both outcome and explanatory) collected for the year 2014 or closest approximation were selected as the GSNI collection period ranged from 2005-2014. This included CVD mortality, GDP per capita, physicians per 1000 population, mean years of schooling and maternal mortality ratio. Life expectancy data from 2015 was used due to availability. If a value was not available for the year of analysis for one of the explanatory variables, a value for the most recent year available was inserted. If years either side of the missing year were available, the most contemporaneous year was selected.

*Statistical analysis*

Normally distributed continuous variables were summarised using mean and standard deviation. If not normally distributed, variables were summarised using median and inter-quartile ranges. Categorical variables were summarised using frequency tables. The GSNI


For peer review only - http://bmjopen.bmj.com/site/about/guidelines.xhtml

values were compared over time for countries with values for both collection waves, and the values with compared between collection waves. Differences in these groups were assessed visually with the use of box plots and statistically using appropriate parametric or non-parametric hypothesis tests after assessing the normality and variance of the data.

Scatter plots were used to visualise the relationships between each of the outcome and explanatory variables. If the relationship was not linear, consideration of variable transformation was made for further analysis.

Univariable linear regression models were computed for each of the 3 outcome variables and each of the explanatory variables in turn. Multivariable linear regression models were then created for each outcome variable using the ordinary least squares method[35]. Testing of the null hypothesis ($\beta_1 = 0$) was done with use of the $t$-distribution where $t = \beta / SE$. Where SE is the standard error of the slope. Model goodness-of-fit was assessed with use of $R^2$ and the F statistic[35, 36].

To act as a sensitivity analysis to the choice of data time points used, the analysis was repeated for outcome and covariate data collected for 2017 (2019 for life expectancy) as this is the most recent year for which the majority of data points were available. Sensitivity analysis was also conducted with the use of GSNI1 values to test the choice primary explanatory variable.

The assumptions for linear regression models (linearity, collinearity, homoscedasticity and normality of errors) were assessed with use of appropriate plots. Residuals vs leverage plots and Cook's distance were used to identify potential outliers or influential observations.


For peer review only - http://bmjopen.bmj.com/site/about/guidelines.xhtml
For peer review only

The significance level for p-values was set at 0.05 for all statistical tests. The statistical assumptions required for linear regression models were tested and deemed acceptable for all models. All data management and analysis were conducted using R version 4.1.2.

*Patient and public involvement*

No members of the public or patients were involved in the design or implementation of the study.

## Results

Summary statistics for the 75 countries with Gender Social Norms Index values available can be viewed in *table 1*. Female age-standardised CVD mortality rate ranged from 62 per 100 000 to 1025 per 100 000 (IQR 128 to 345). Male age-standardised CVD mortality rate ranged from 107 per 100 000 to 1205 per 100 000 (IQR 180 to 429). Age-standardised CVD mortality for the total population ranged from 83 to 1107 per 100 000 (IQR 156 to 373). Japan had the lowest male, female and total CVD mortality rates and Uzbekistan the highest. The ratio of female to male CVD mortality rates ranged from 0.5 in Viet Nam to 1.6 in Qatar (IQR 0.67 to 0.85). Female life expectancy ranged from 61 to 86.4 years (IQR 74.5 to 82.6). Zimbabwe had the lowest female life expectancy and Japan the highest. Male life expectancy ranged from 55.7 years in Zimbabwe to 80.7 years in both Japan and Singapore (IQR 67.9 to 77.6).

The GSNI value for 2 or more biases ranged from 7.4% in Andorra to 98.1% in Pakistan (median 68.6, IQR 41.9 to 86.3). 31 countries had a GSNI2 value available for both collection periods, with no statistically significant difference between the values across the two time periods (paired t-test = 0.104, p-value 0.92) (*supplemental material figure S1*).


For peer review only - http://bmjopen.bmj.com/site/about/guidelines.xhtml

Comparison was made between the GSNI2 values included in the analysis (n=75) collected for each time period (*supplemental material figure S1*). Those collected during the later period were statistically significantly higher than those collected earlier (Man-Whitney U test = 320, p-value = 0.02).

| **Summary Statistics** | **n = 75** |
|---|---|
| **Female CVD age adjusted mortality rate (per 100 000 population)**<br>Year: 2014<br>Source: Global Burden of Disease 2019 | |
| min | 62 |
| max | 1024.9 |
| median (IQR) | 241.30 (128.06, 345.30) |
| missing | 0 |
| **Male CVD age adjusted mortality rate (per 100 000 population)**<br>Year: 2014<br>Source: Global Burden of Disease 2019 | |
| min | 106.8 |
| max | 1205.2 |
| median (IQR) | 278.05 (180.32, 428.51) |
| missing | 0 |
| **Population CVD age adjusted mortality rate (per 100 000 population)**<br>Year: 2014<br>Source: Global Burden of Disease 2019 | |
| min | 82.6 |
| max | 1107 |
| median (IQR) | 262.98 (155.95, 372.59) |
| missing | 0 |
| **Ratio of female to male CVD mortality rates**<br>Year: 2014<br>Source: Global Burden of Disease 2019 | |
| min | 0.5 |
| max | 1.6 |
| median (IQR) | 0.72 (0.67, 0.85) |
| missing | 0 |


For peer review only - http://bmjopen.bmj.com/site/about/guidelines.xhtml

| | |
|---|---|
| **Female life expectancy at birth**<br>Year: 2015<br>Source: World Health Observatory | |
| min | 61 |
| max | 86.4 |
| median (IQR) | 78.60 (74.45, 82.60) |
| missing | 0 |
| **Male life expectancy at birth**<br>Year: 2015<br>Source: World Health Observatory | |
| min | 55.7 |
| max | 80.7 |
| median (IQR) | 73.00 (67.90, 77.60) |
| missing | 0 |
| **GSNI index 2 or more bias**<br>Year: 2005-2014<br>Source: Gender Social Norms Index | |
| min | 7.4 |
| max | 98.1 |
| median (IQR) | 68.56 (41.94, 86.33) |
| missing | 0 |
| **Year of Index Collection**<br>Source: Gender Social Norms Index | |
| 2005-2009 | 18 (24) |
| 2010-2014 | 57 (76) |
| **Physicians per 1000 population**<br>Year: 2014<br>Source: World Bank | |
| min | 0 |
| max | 5 |
| median (IQR) | 71; 2.30 (1.29, 3.22) |
| missing | 4 |
| **GDP per capita**<br>Year: 2014<br>Source: World Bank | |
| min | 566.9 |
| max | 97019.2 |
| median (IQR) | 68; 9181 (4094, 31623) |
| missing | 7 |


For peer review only - http://bmjopen.bmj.com/site/about/guidelines.xhtml

| | |
|---|---|
| **Maternal mortality ratio**<br>**Year: 2014**<br>**Source: World Bank** | |
| min | 2 |
| max | 943 |
| median (IQR) | 70; 25.50 (10.25, 82.75) |
| missing | 5 |
| | |
| **Mean years of schooling**<br>**Year: 2014**<br>**Source: Our World In Data** | |
| min | 1.4 |
| max | 14 |
| median (IQR) | 69; 10.10 (7.60, 11.80) |
| missing | 6 |

*Table 1.* Summary statistics. CVD – Cardiovascular disease; IQR – inter-quartile range; GSNI – Gender Social Norms Index; GDP – Gross Domestic Product

GSNI2 was positively correlated with female CVD mortality rates (rho = 0.67, p-value <0.05), with male CVD mortality rates (rho 0.53, p-value <0.05) and with population CVD mortality rates (rho = 0.60, p-value <0.05). GSNI2 was also positively correlated with female to male CVD mortality ratio (rho = 0.57, p-value <0.05) (*figure 1*). GSNI2 was negatively correlated with female life expectancy (rho = -0.72, p-value <0.05) and with male life expectancy (rho = -0.61, p-value = <0.05) (*figure 2*). Please see *supplemental material table S1 and figures S2-3* for the list of countries included and additional scatter plot versions with points labelled by country.

75 countries had data available for the univariable regression analyses. 67 Countries had data available across all covariates for the multivariable regression analyses. Andorra was not included in the female life expectancy outcome as maternal mortality ratio not available (n = 66); Qatar was excluded from the CVD ratio outcome analysis because it was identified as an


For peer review only - http://bmjopen.bmj.com/site/about/guidelines.xhtml

influential outlier (n = 66). The list of included countries within each analysis can be found in *supplemental material tables S1-2.*

In the univariable models, higher levels of gender bias as measured by the GSNI2 were statistically significantly associated with higher female CVD mortality rates ($\beta$ 3.63, 95% CIs 2.48 to 4.78), male CVD mortality rates ($\beta$ 3.57, 95% CIs 2.03 to 5.12) and population CVD mortality rates ($\beta$ 3.57, 95% CIs 2.29 to 4.85). The statistically significant association remained after adjusting for physicians per 1000 population, mean years of schooling, GDP per capita and GSNI data collection period (*table 2 & 3*). The multivariable model results indicate that for every 1% greater proportion of the population who hold 2 or more gender biases, the female CVD mortality rate was higher by 4.86 per 100 000 (95% CI 3.18 to 6.54), male CVD mortality rate higher by 5.28 per 100 000 (95% CI 3.42 to 7.15) and the population CVD mortality rate was higher by 4.89 per 100 000 (95% CI 3.18 to 6.60). In the univariable model GSNI2 was statistically significantly associated with female to male CVD mortality ratio ($\beta$ 0.003, 95% CIs 0.002 to 0.005). This relationship was not significant within the multivariable model ($\beta$ 0.001, 95% CIs -0.001 to 0.003).

*Table 4* demonstrates the model results for the life expectancy outcomes. In the univariable analysis, higher levels of gender bias as measured by the GSNI2 were statistically significantly associated with lower female life expectancy ($\beta$ -0.16, 95% CIs -0.20 to -0.12) and lower male life expectancy ($\beta$ -0.15, 95% CIs -0.20 to -0.11). This relationship remained statistically significant after controlling for physicians per 1000 population, mean years of schooling, GDP per capita and GSNI data collection period. Maternal mortality ratio was also included within the female life expectancy model. Within the life expectancy multivariable models, the physicians per 1000 and GDP per capita variables were log transformed due to


For peer review only - http://bmjopen.bmj.com/site/about/guidelines.xhtml

their relationship with the outcome variable. In the final life expectancy model the coefficient indicates that for every 1% increase in the GSNI2, female life expectancy was lower by 0.07 years (25.6 days) (95% CI -0.11 to -0.03) and male life expectancy was lower by 0.05 years (18.3 days) (95% CI -0.10 to -0.01).

The sensitivity analysis made use of data from 2017 and 2019 with no change in direction of association between each outcome variable and GSNI2. The only change in statistical significance compared to the primary analysis was the multivariable male life expectancy model in which male life expectancy was not found to be statistically significantly associated with GSNI2 (*supplemental material tables S3-5*). GSNI1 and GSNI2 were strongly positively correlated (rho = 0.98, p-value = <0.05) and the results of sensitivity analysis demonstrated no change in interpretation of results with use of GSNI1 variable as an alternative to GSNI2. The results of this analysis are available on request.

For peer review only

| | *Dependent variable:* | | | |
|---|---|---|---|---|
| | female CVD mortality 2014 | | male CVD mortality 2014 | |
| | (1) | (2) | (3) | (4) |
| constant | 30.04 (39.25) | -133.15 (94.46) | 108.45* (52.87) | -184.13 (104.74) |
| GSNI2 | 3.63*** (0.58) | 4.86*** (0.84) | 3.57*** (0.78) | 5.28*** (0.93) |
| physicians per 1000 | | 32.31 (19.26) | | 70.59** (21.35) |
| mean years of schooling | | 13.75 (9.37) | | 21.71* (10.39) |
| GDP per capita | | -0.002* (0.001) | | -0.004*** (0.001) |
| GSNI data collection period 2010-2014 | | -97.89* (40.74) | | -127.18** (45.17) |
| $R^2$ | 0.35 | 0.49 | 0.23 | 0.56 |
| Adjusted $R^2$ | 0.34 | 0.44 | 0.21 | 0.53 |
| F Statistic | 39.68*** (df = 1; 73) | 11.54*** (df = 5; 61) | 21.23*** (df = 1; 73) | 15.62*** (df = 5; 61) |

* p<0.05, **p<0.01,***p<0.001

*Table 2.* Results of univariable and multivariable regression models for the female and male CVD mortality outcomes. Numbers in brackets are standard errors.

GSNI2 – Gender Social Norms Index 2; GDP – Gross Domestic Product; CVD – Cardiovascular disease

For peer review only

| | *Dependent variable:* | | | |
|---|---|---|---|---|
| | CVD mortality 2014 | | female to male CVD mortality ratio 2014 | |
| | (1) | (2) | (3) | (4) |
| constant | 65.58 (43.71) | -140.69 (95.92) | 0.57*** (0.05) | 0.90*** (0.09) |
| GSNI2 | 3.57*** (0.64) | 4.89*** (0.85) | 0.003*** (0.001) | 0.001 (0.001) |
| physicians per 1000 | | 47.45* (19.55) | | -0.04* (0.02) |
| mean years of schooling | | 17.25 (9.52) | | -0.01 (0.01) |
| GDP per capita | | -0.003** (0.001) | | -0.0000 (0.0000) |
| GSNI data collection period 2010-2014 | | -110.79** (41.36) | | -0.03 (0.04) |
| $R^2$ | 0.30 | 0.53 | 0.24 | 0.46 |
| Adjusted $R^2$ | 0.29 | 0.49 | 0.22 | 0.42 |
| F Statistic | 30.97*** (df = 1; 73) | 13.54*** (df = 5; 61) | 22.44*** (df = 1; 73) | 10.30*** (df = 5; 60) |

* p<0.05, **p<0.01,***p<0.001

***Table 3.*** Results of univariable and multivariable regression models for CVD mortality and female to male CVD mortality ratio. Numbers in brackets are standard errors.

GSNI2 – Gender Social Norms Index 2; GDP – Gross Domestic Product; CVD – Cardiovascular disease



For peer review only - http://bmjopen.bmj.com/site/about/guidelines.xhtml

| | *Dependent variable:* | | | |
|---|---|---|---|---|
| | female life expectancy 2015 | | male life expectancy 2015 | |
| | (1) | (2) | (3) | (4) |
| constant | $87.31^{***}$ (1.46) | $68.74^{***}$ (4.82) | $81.65^{***}$ (1.51) | $50.48^{***}$ (5.98) |
| GSNI | $-0.16^{***}$ (0.02) | $-0.07^{***}$ (0.02) | $-0.15^{***}$ (0.02) | $-0.05^{*}$ (0.02) |
| log (physicians per 1000) | | $1.27^{*}$ (0.61) | | $1.95^{**}$ (0.66) |
| mean years of schooling | | -0.41 (0.21) | | $-0.69^{*}$ (0.27) |
| log (GDP per capita) | | $1.82^{***}$ (0.46) | | $3.29^{***}$ (0.58) |
| GSNI data collection period 2010-2014 | | 1.30 (0.91) | | 0.95 (1.14) |
| MMR | | $-0.01^{***}$ (0.003) | | |
| $R^2$ | 0.43 | 0.86 | 0.39 | 0.76 |
| Adjusted $R^2$ | 0.42 | 0.84 | 0.38 | 0.74 |
| F Statistic | $55.39^{***}$ (df = 1; 73) | $60.02^{***}$ (df = 6; 59) | $46.59^{***}$ (df = 1; 73) | $39.35^{***}$ (df = 5; 61) |

* p<0.05, **p<0.01,***p<0.001

*Table 4.* Results of univariable and multivariable regression models for female life expectancy at birth and male life expectancy at birth. Numbers in brackets are standard errors.

GSNI2 – Gender Social Norms Index 2; GDP – Gross Domestic Product; CVD – Cardiovascular disease; MMR – Maternal mortality ratio

## Discussion

We found a statistically significant relationship between higher country levels of biased gender social norms as measured by the Gender Social Norms Index and higher CVD mortality rates and lower life expectancy for both females and males. These results remained statistically significant after adjusting for country-level economic, education, and healthcare status measures. There was no statistically significant relationship demonstrated between the Gender Social Norms Index and female to male CVD mortality ratios within the adjusted model.

Despite a considerable body of research on the relationship between gender inequalities and health, there has been little to no previous quantitative investigation into how gender social norms may link to CVD and life expectancy. Kim *et al* have linked greater gender inequality


For peer review only - http://bmjopen.bmj.com/site/about/guidelines.xhtml

as measured by the Gender Inequality Index to higher female to male stroke mortality ratios and lower life expectancy, with use of similar ecological study designs[37-40]. However, some of these analyses did not control for confounding factors leaving results open to significant bias. The choice of covariates used in our study were designed to control for likely confounders within the realms of economics, education and healthcare provision at the country level, all known to have relationships with population health[28-30].

Additionally, previous research in the area of gender inequality and health has made use of the Gender Inequality Index as the primary explanatory variable[37-40]. However, linking this particular index to health outcomes is problematic as the Gender Inequality Index includes maternal mortality ratio, which is inherently related to health outcomes. The use of the Gender Social Norms Index within our study avoids this limitation. Furthermore, the Gender Inequality Index is a measure of the outcomes of gender inequality e.g. adolescent birth rates and female shares of parliamentary seats, rather than a measure of the social norms held by populations which influence these unequal outcomes, this study aimed to explore the latter.

O'Neil *et al.* have proposed pathways through which gender influences norms, roles, attitudes and knowledge to affect exposure to psychosocial stressors and health behaviours which influence risk of CVD. For example, there is evidence that boys are socialised to take up more physical activity compared with girls, and adolescent girls are more likely to use smoking as a weight loss tool due to social pressures[19]. Additionally, the authors argue that traditional gender roles at home and work may be associated with worse cardiovascular health, including the expectations for men to be the sole breadwinner and women to take on care giving responsibilities[19]. The psychological impact of sexual abuse and gender discrimination has also been identified as a likely risk factor for CVD[19]. The results of our



For peer review only - http://bmjopen.bmj.com/site/about/guidelines.xhtml

For peer review only

study support this theoretical framework with the suggestion at a country level of a significant association between gender social norms and population-level CVD outcomes and life expectancy.

*Strengths and limitations*

The strengths of this study include the robustness of the underlying data used, its global coverage, and novel use of the Gender Social Norms Index. Additionally, important potentially confounding factors have been adjusted for within the multivariable models. Sensitivity analysis was conducted demonstrating that all results were consistent across differing data time points apart from the male life expectancy outcome.

There are, however, important limitations to consider when interpreting the results of this analysis. Gender social norms are a complex social phenomenon and therefore challenging to measure and quantify, and the underlying data used to calculate the GSNI will likely be affected by selection bias, non-response bias and social desirability bias. Additionally, GSNI values were not available for all global countries.

The GSNI results included in this study were collected over a long period of time (2005-2014) and therefore there may be lack of standardisation between the data collected at different time points. It is hoped that including the GSNI collection period within the multivariable model will adjust for any systematic differences. However, whilst the GSNI values were not found to be statistically significantly different over time between the 2 waves of data collection, only 31 countries had data available for both time points. It may be the case that a country which had GSNI data collected in 2005 may have significantly changed norms in 2014, which is the time point used for the other data points within the main analysis. The sensitivity analysis ran the model with data from 2017, the results of which did not change in direction or significance apart from the male life expectancy outcome. However, it is noted that if GSNI data was collected in 2005 that remains over a decade in which gender social norms may have changed.


For peer review only - http://bmjopen.bmj.com/site/about/guidelines.xhtml

Ecological studies are utilised to measure population level variables and to influence policy at a regional and national scale[41]. The recognition of gender social norms as pervasive in the environment and influential beyond the individual was deemed an appropriate design for this research question. However, it is well recognised that associations at the ecological level may not be seen at an individual level[42]. It could be argued that understanding the relationship between individually held gender social norms and individual health is an important but different question. However, the results of this research must be interpreted with caution in this regard.

Finally, the variables used as covariates with an unclear causal pathway may have introduced bias into the models. It is noted that the models demonstrate potentially unexpected covariate results including higher mean years of schooling and higher numbers of physicians per population associated with higher CVD mortality. This may be the result of countries with improving levels of such determinants experiencing increased burden from non-communicable diseases like CVD due to increased survival from other causes. As with any ecological study, it is essential to recognise this design cannot lead to any firm conclusions about a causal relationship between variables. However, our results suggest a strong positive and statistically significant association between gender social norms and CVD mortality that merits further exploration.

*Implications*

The results of this research imply there is an association between country level gender social norms and CVD mortality and life expectancy for males and females. The lack of association between gender social norms and female to male CVD mortality ratio suggests that gender


For peer review only - http://bmjopen.bmj.com/site/about/guidelines.xhtml

social norms may affect both male and female CVD mortality rates equally. Based on the modelling results, a 10% improvement in a country's Gender Social Norms Index score could be associated with a 50 per 100 000 reduction in CVD mortality rates for both males and females. Additionally, the total variation observed between countries in Gender Social Norm Index value could account for 6.3 years of life expectancy difference in females and 4.5 years in males. This supports the hypothesis that gender social norms are a social determinant of CVD and overall health more broadly. It is likely that there are additional pathways at play affecting the relationship between gender social norms and life expectancy besides its association with CVD mortality and this warrants further investigation.

The relationships between gender social norms and CVD outcomes and life expectancy should be explored further with use of alternative measures of gender social norms, analysis over time and explore options for mediation analysis on the individual-level. Country case studies could also be explored for those with extreme values of the Gender Social Norms Index. The findings of this research suggest that it would be justified to investigate biased gender social norms as a phenomenon that may impact negatively on the entire population's health, in wide ranging ways, particularly in the area of non-communicable diseases. Therefore, this research should be added to the ever growing body of evidence that improving biased gender social norms and gender equality can contribute to better population health and should be a priority for policy decision makers as set out within the Sustainable Development Goals.


For peer review only - http://bmjopen.bmj.com/site/about/guidelines.xhtml

Glossary Box

***Sex*** **-** Biologically determined characteristics (genetics, hormones, anatomy) that lead to categorisation of individuals, typically into male, female and intersex.

***Gender*** **-** Considered a social construct, sociologically determined understandings and behaviours interacting with but separate from sex. Influenced by societal norms and changes over time.

***Social norms*** **-** The unwritten societal expectations influencing how we think and behave in a social situation, tend to be considered on an individual level i.e. how individuals react to what societal expectations exist.

***Gender norms*** **-** Engrained understandings of how individuals should act based on their gender, learnt through institutions such as family, school, workplace, religion, media and other social institutions. Conceptualised as a force external to individuals, upheld by institutions. Contributes to the perpetuation of the gender system which on the whole tends to favour males and masculinity.


For peer review only - http://bmjopen.bmj.com/site/about/guidelines.xhtml

**Contributors**:

IL, AH and MOF conceived and designed the study. IL acquired and managed the data and completed all statistical analysis, which was reviewed by AH and MOF. IL wrote the first draft and is the guarantor for this study. SK and ML contributed to the critical discussion of the results and manuscript drafting. The corresponding author attests that all listed authors meet authorship criteria and that no others meeting the criteria have been omitted.

**Copyright:**

The Corresponding Author has the right to grant on behalf of all authors and does grant on behalf of all authors, a worldwide licence to the Publishers and its licensees in perpetuity, in all forms, formats and media (whether known now or created in the future), to i) publish, reproduce, distribute, display and store the Contribution, ii) translate the Contribution into other languages, create adaptations, reprints, include within collections and create summaries, extracts and/or, abstracts of the Contribution, iii) create any other derivative work(s) based on the Contribution, iv) to exploit all subsidiary rights in the Contribution, v) the inclusion of electronic links from the Contribution to third party material where-ever it may be located; and, vi) licence any third party to do any or all of the above.

For peer review only

**Funding:**

IL is an Academic Clinical Fellow funded by the UK National Institute for Health and Care Research in conjunction with Lancaster University. They are employed by St Helens and Knowsley NHS Foundation Trust.



For peer review only - http://bmjopen.bmj.com/site/about/guidelines.xhtml

AH is funded by a departmental studentship at the University of Liverpool and supported by the UK National Institute for Health and Care Research School for Public Health (Grant Reference Number PD-SPH-2015-10025).

SK receives funding from the National Heart, Lung and Blood Institute and the American Heart Association.

These funders had no role in the study design, analysis or presentation of results. The views expressed are those of the authors and not necessarily those of the National Institute for Health and Care Research, the NHS, the National Heart, Lung and Blood Institute or the American Heart Association.

Prof Martin O'Flaherty is partly funded (among other grants) by ESRC grant ES/W007932/1: Developing public health policies for the out of home food sector to improve diet and reduce obesity, and NIHR grant NIHR130258: Unmet need for healthcare.

**Competing interests:**

All authors have completed the ICMJE uniform disclosure form at www.icmje.org/coi_disclosure.pdf and declare: no support from any organisation for the submitted work; no financial relationships with any organisations that might have an interest in the submitted work in the previous three years; no other relationships or activities that could appear to have influenced the submitted work.

**Ethical approval:** All data used in this research was non-identifiable and freely available. Therefore, after submission to the University of Liverpool ethics review board (reference

For peer review only

number 9811) the research was approved without the need for review by the ethics committee.

**Transparency statement**: The manuscript's lead author and guarantor (IL) affirms that the manuscript is an honest, accurate, and transparent account of the study being reported; no important aspects of the study have been omitted and any discrepancies from the study as originally planned have been explained.

**Data sharing:** The data used in this analysis are openly available from the Institute for Health Metrics and Evaluation, The World Bank, The Global Health Observatory, the United Nations Development Programme and Our World in Data. Code used for analysis is available via GitHub here.

**Liscence statement:** I, the Submitting Author has the right to grant and does grant on behalf of all authors of the Work (as defined in the author licence), an exclusive licence and/or a non-exclusive licence for contributions from authors who are: i) UK Crown employees; ii) where BMJ has agreed a CC-BY licence shall apply, and/or iii) in accordance with the terms applicable for US Federal Government officers or employees acting as part of their official duties; on a worldwide, perpetual, irrevocable, royalty-free basis to BMJ Publishing Group Ltd ("BMJ") its licensees.

The Submitting Author accepts and understands that any supply made under these terms is made by BMJ to the Submitting Author unless you are acting as an employee on behalf of your employer or a postgraduate student of an affiliated institution which is paying any



For peer review only - http://bmjopen.bmj.com/site/about/guidelines.xhtml

applicable article publishing charge ("APC") for Open Access articles. Where the Submitting Author wishes to make the Work available on an Open Access basis (and intends to pay the relevant APC), the terms of reuse of such Open Access shall be governed by a Creative Commons licence – details of these licences and which licence will apply to this Work are set out in our licence referred to above.

**References**

1. United Nations. The Sustainable Development Goals Report 2020. 2020. United Nations. https://unstats.un.org/sdgs/report/2020/The-Sustainable-Development-Goals-Report-2020.pdf

2. Clark J and Horton R. A coming of age for gender in global health. The Lancet 2019; 393: 2367-2369.

3. Hay K, McDougal L, Percival V, et al. Disrupting gender norms in health systems: making the case for change. The Lancet 2019; 393: 2535-2549.

4. Heise L, Greene ME, Opper N, et al. Gender inequality and restrictive gender norms: framing the challenges to health. The Lancet 2019; 393: 2440-2454.

5. Heymann J, Levy JK, Bose B, et al. Improving health with programmatic, legal, and policy approaches to reduce gender inequality and change restrictive gender norms. The Lancet 2019; 393: 2522-2534.

6. Weber AM, Cislaghi B, Meausoone V, et al. Gender norms and health: insights from global survey data. The Lancet 2019; 393: 2455-2468.

7. Mejía-Guevara I, Cislaghi B, Weber A, et al. Association of collective attitudes and contraceptive practice in nine sub-Saharan African countries. Journal of global health 2020; 10.

8. Shakya HB, Darmstadt GL, Barker KM, et al. Social normative and social network factors associated with adolescent pregnancy: a cross-sectional study of 176 villages in rural Honduras. Journal of global health 2020; 10: 010706-010706.

9. Stark L, Seff I, Weber AM, et al. Perpetration of intimate partner violence and mental health outcomes: sex- and gender-disaggregated associations among adolescents and young adults in Nigeria. Journal of global health 2020; 10: 010708-010708.



For peer review only - http://bmjopen.bmj.com/site/about/guidelines.xhtml

10. Omidakhsh N and Heymann J. Improved child marriage laws and its association with changing attitudes and experiences of intimate partner violence: a comparative multi-national study. Journal of global health 2020; 10: 010707-010707.

11. Bonita R and Beaglehole R. Women and NCDs: overcoming the neglect. Global health action 2014; 7: 23742. 2014/05/09.

12. Homan P. Political gender inequality and infant mortality in the United States, 1990–2012. Social Science & Medicine 2017; 182: 127-135.

13. Heaton TB. Are Improvements in Child Health Due to Increasing Status of Women in Developing Nations? Biodemography and Social Biology 2015; 61: 252-265.

14. Stark L, Seff I, Weber A, et al. Applying a gender lens to global health and well-being: Framing a Journal of Global Health special collection. Journal of global health 2020; 10: 010103. 2020/04/08.

15. Vos T, Lim SS, Abbafati C, et al. Global burden of 369 diseases and injuries in 204 countries and territories, 1990–2019: a systematic analysis for the Global Burden of Disease Study 2019. The Lancet 2020; 396: 1204-1222.

16. World Health Organisation. 2021. Gender and Health, https://www.who.int/health-topics/gender#tab=tab_1 (accessed 09/07/2021).

17. Kim ES and Menon V. Status of women in cardiovascular clinical trials. Arteriosclerosis, thrombosis, and vascular biology 2009; 29: 279-283.

18. Cislaghi B and Heise L. Gender norms and social norms: differences, similarities and why they matter in prevention science. Sociology of health & illness 2020; 42: 407-422. https://doi.org/10.1111/1467-9566.13008.

19. O'Neil A, Scovelle AJ, Milner AJ, et al. Gender/Sex as a Social Determinant of Cardiovascular Risk. Circulation 2018; 137: 854-864.

20. Walli-Attaei M, Joseph P, Rosengren A, et al. Variations between women and men in risk factors, treatments, cardiovascular disease incidence, and death in 27 high-income, middle-income, and low-income countries (PURE): a prospective cohort study. The Lancet 2020; 396: 97-109.

21. Liakos M and Parikh PB. Gender Disparities in Presentation, Management, and Outcomes of Acute Myocardial Infarction. Current Cardiology Reports 2018; 20: 64.

22. Woodward M. Cardiovascular Disease and the Female Disadvantage. International Journal of Environmental Research and Public Health 2019; 16 2019/04/04.

23. Wang H, Abbas KM, Abbasifard M, et al. Global age-sex-specific fertility, mortality, healthy life expectancy (HALE), and population estimates in 204 countries and territories, 1950–2019: a comprehensive demographic analysis for the Global Burden of Disease Study 2019. The Lancet 2020; 396: 1160-1203.

For peer review only

24. The Global Health Observatory. 2021. Life expectancy at birth (years), https://www.who.int/data/gho/data/indicators/indicator-details/GHO/life-expectancy-at-birth-(years) (accessed 14/07/2021).

25. Institute for Health Metrics and Evaluation. 2021. GBD Compare, https://vizhub.healthdata.org/gbd-compare/le (accessed 07/07/2021).

26. Institute for Future Studies. 2021. World Values Survey, https://www.iffs.se/en/world-values-survey/ (accessed 14/07/2021).

27. United Nations Development Programme. Frequenty Asked Questions - Gender Social Norms Index (GSNI). 2020. http://hdr.undp.org/sites/default/files/frequently_asked_questions_gsni.pdf

28. Swift R. The relationship between health and GDP in OECD countries in the very long run. Health Economics 2011; 20: 306-322.

29. Cutler DM and Lleras-Muney A. Education and health: insights from international comparisons. 2012.

30. Basu S, Berkowitz SA, Phillips RL, et al. Association of Primary Care Physician Supply With Population Mortality in the United States, 2005-2015. JAMA Internal Medicine 2019; 179: 506-514.

31. The World Bank. 2021. GDP per capita (current US$), https://data.worldbank.org/indicator/NY.GDP.PCAP.CD (accessed 01/06/2021).

32. The World Bank. 2021. Physicians (per 1,000 people), https://data.worldbank.org/indicator/SH.MED.PHYS.ZS (accessed 01/06/2021).

33. Roser M and Ortiz-Ospina E. 2016. Global Education, https://ourworldindata.org/global-education (accessed 03/06/2021).

34. The World Bank. 2021. Maternal mortality ratio (modeled estimate, per 100,000 live births), https://data.worldbank.org/indicator/SH.STA.MMRT (accessed 01/06/2021).

35. Bruce N, Pope D and Stanistreet D. Quantitative methods for health research. Wiley Online Library, 2017. 1118665376.

36. Leach LF and Henson RK. The use and impact of adjusted R2 effects in published regression research. Multiple Linear Regression Viewpoints 2007; 33: 1-11.

37. Kim JI and Kim G. Labor force participation and secondary education of gender inequality index (GII) associated with healthy life expectancy (HLE) at birth. International Journal for Equity in Health 2014; 13: 106.

38. Kim JI and Kim G. Socio-ecological perspective of older age life expectancy: income, gender inequality, and financial crisis in Europe. Globalization & Health 2017; 13: 1-8.

39. Kim JI and Kim G. Country-level socioeconomic indicators associated with survival probability of becoming a centenarian among older european adults: gender inequality, male



For peer review only - http://bmjopen.bmj.com/site/about/guidelines.xhtml

labour force participation and proportions of women in parliaments. Journal of biosocial science 2017; 49: 239-250.

40. Kim YD, Jung YH, Caso V, et al. Countries with women inequalities have higher stroke mortality. International journal of stroke : official journal of the International Stroke Society 2017; 12: 869-874.

41. Adeyinka DA, Evans MR, Ozigbu CE, et al. Understanding the Influence of Socioeconomic Environment on Paediatric Antiretroviral Treatment Coverage: Towards Closing Treatment Gaps in Sub-Saharan Africa. Central European journal of public health 2017; 25: 55-63.

42. Sedgwick P. Ecological studies: advantages and disadvantages. BMJ 2014; 348: g2979.

For peer review only

## Figure legend

**Figure 1.** Scatter plots of GSNI2 against female, male and population age standardised CVD mortality rates. (GSNI2 – Gender Social Norms Index 2; CVD – Cardiovascular disease)

**Figure 2.** Scatter plots of GSNI2 against female and male life expectancy. (GSNI2 – Gender Social Norms Index 2)

For peer review only


For peer review only - http://bmjopen.bmj.com/site/about/guidelines.xhtml

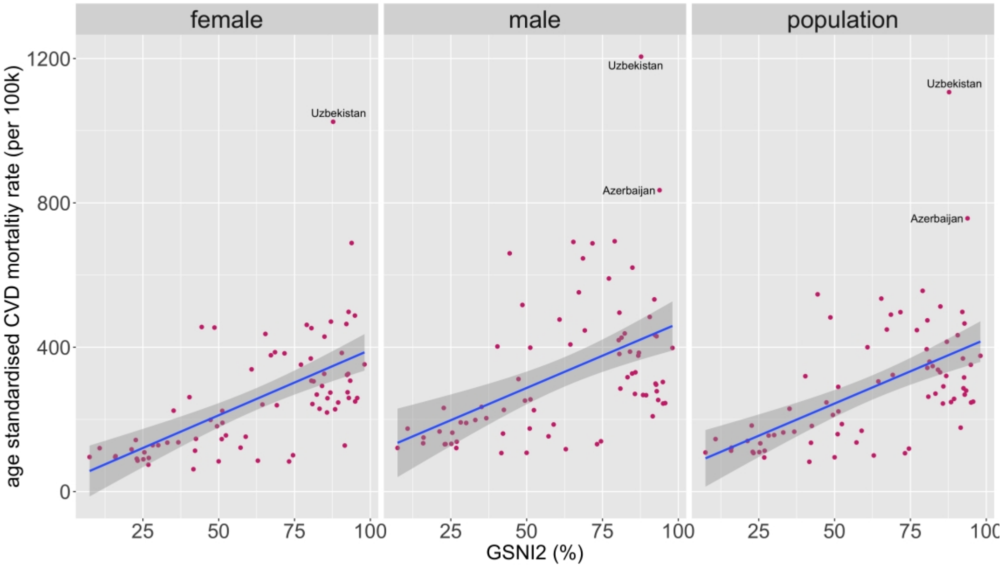


Figure 1. Scatter plots of GSNI2 against female, male and population age standardised CVD mortality rates. (GSNI2 – Gender Social Norms Index 2; CVD – Cardiovascular disease)

1458x833mm (72 x 72 DPI)

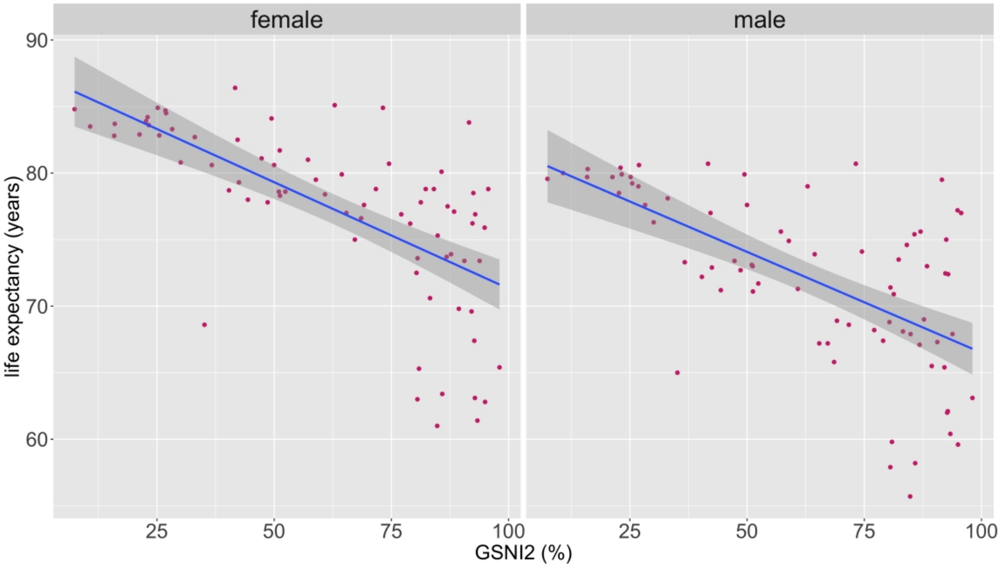


Figure 2. Scatter plots of GSNI2 against female and male life expectancy. (GSNI2 – Gender Social Norms Index 2)

1458x833mm (72 x 72 DPI)

Supplemental Material:

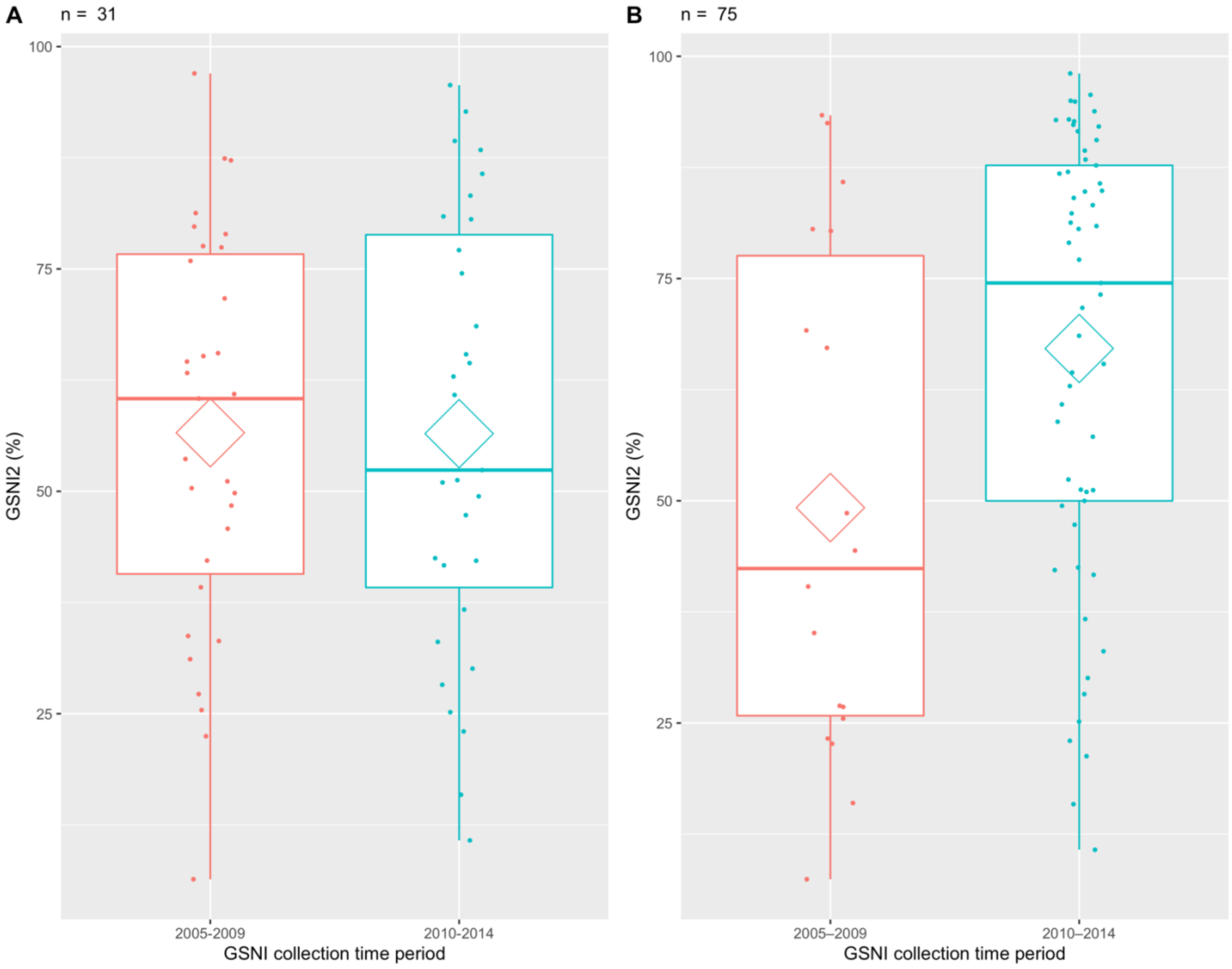


**Figure S1. A.** Box plots comparing GSNI2 values between countries that had values for both 2005-2009 collection period and 2010-2014 collection period. **B.** Box plots splitting the GSNI2 values included in the analysis by period of collection.

**Table S1.** List of countries used within scatter plots (figure 1 and 2) and univariable analyses
n=75

| | | |
|---|---|---|
| Algeria | India | Poland |
| Andorra | Indonesia | Qatar |
| Argentina | Iran, Islamic Republic of | Romania |
| Armenia | Iraq | Russian Federation |
| Australia | Japan | Rwanda |
| Azerbaijan | Jordan | Serbia |
| Belarus | Kazakhstan | Singapore |
| Brazil | Korea (Republic of) | Slovenia |
| Bulgaria | Kuwait | South Africa |
| Burkina Faso | Kyrgyzstan | Spain |
| Canada | Lebanon | Sweden |
| Chile | Libya | Switzerland |
| China | Malaysia | Thailand |
| Colombia | Mali | Trinidad and Tobago |
| Cyprus | Mexico | Tunisia |
| Ecuador | Moldova, Republic of | Turkey |
| Estonia | Morocco | Ukraine |
| Ethiopia | Netherlands | United Kingdom |
| Finland | New Zealand | United States |
| France | Nigeria | Uruguay |
| Georgia | Norway | Uzbekistan |
| Germany | Pakistan | Viet Nam |
| Ghana | Palestine, State of | Yemen |
| Haiti | Peru | Zambia |
| Hungary | Philippines | Zimbabwe |

For peer review only

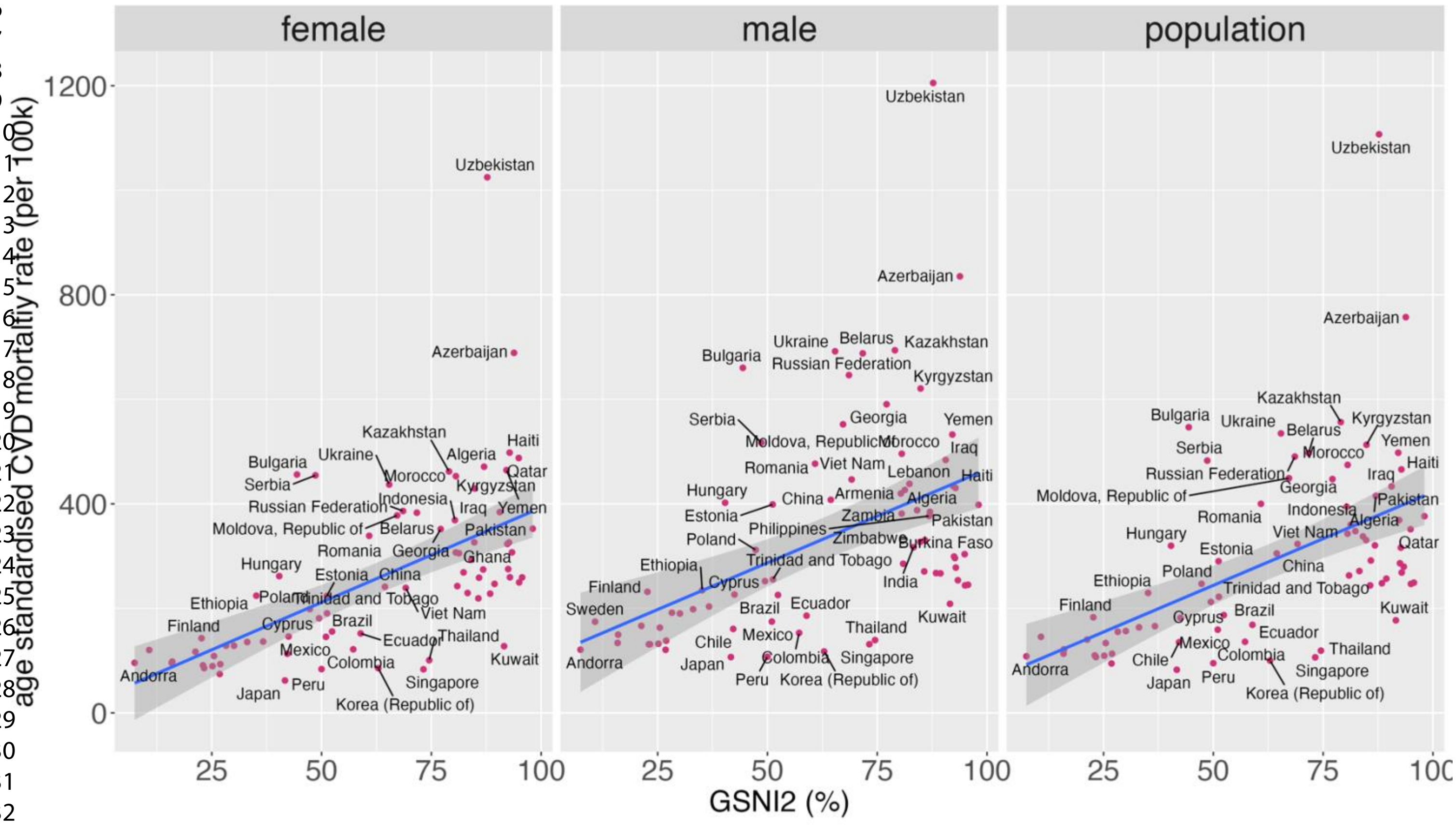


**Figure S2.** Scatter plots of GSNI2 against female, male and population age standardised CVD mortality rates. (GSNI2 – Gender Social Norms Index 2; CVD – Cardiovascular disease. For an interactive version of this graph please visit https://ilsumme.github.io/gsn-cvd-le/

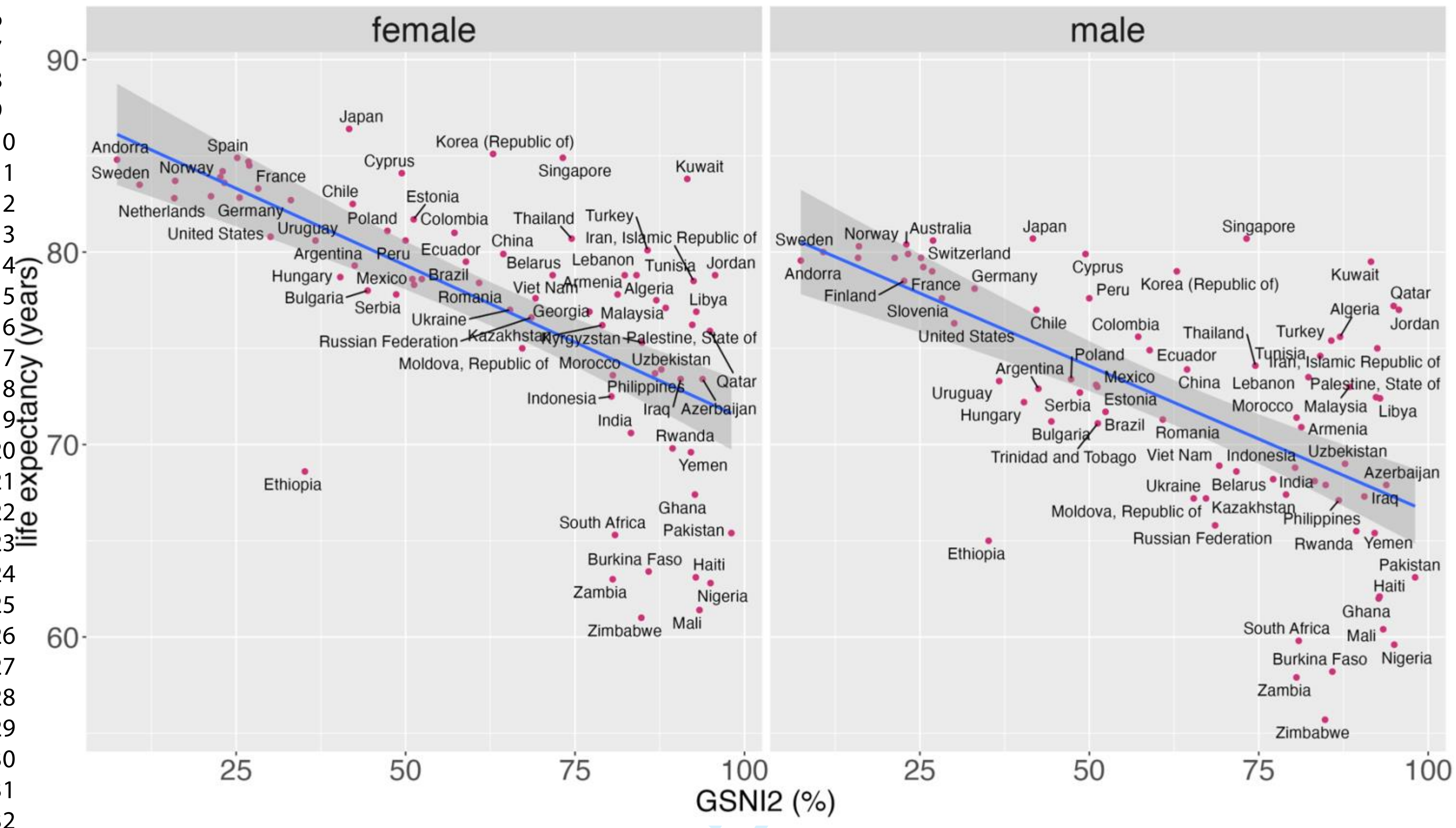


**Figure S3.** Scatter plots of GSNI2 against female and male life expectancy. (GSNI2 – Gender Social Norms Index 2; CVD – Cardiovascular disease. For an interactive version of this graph please visit https://ilsumme.github.io/gsn-cvd-le/

only

**Table S2.** List of countries used within multivariable analyses (Andorra not included in female life expectancy outcome as maternal mortality ratio not available; Qatar not included in CVD ratio outcome as identified as an influential outlier)
n=67

| | | |
|---|---|---|
| Algeria | Ghana | Philippines |
| Andorra | Haiti | Poland |
| Argentina | Hungary | Qatar |
| Armenia | India | Romania |
| Australia | Indonesia | Rwanda |
| Azerbaijan | Iraq | Serbia |
| Belarus | Japan | Singapore |
| Brazil | Jordan | Slovenia |
| Bulgaria | Kazakhstan | South Africa |
| Burkina Faso | Kuwait | Spain |
| Canada | Lebanon | Sweden |
| Chile | Libya | Switzerland |
| China | Malaysia | Thailand |
| Colombia | Mali | Trinidad and Tobago |
| Cyprus | Mexico | Tunisia |
| Ecuador | Morocco | Turkey |
| Estonia | Netherlands | Ukraine |
| Ethiopia | New Zealand | United Kingdom |
| Finland | Nigeria | United States |
| France | Norway | Uruguay |
| Georgia | Pakistan | Uzbekistan |
| Germany | Peru | Zambia |
| | | Zimbabwe |

For peer review only

| | *Dependent variable:* | | | |
|---|---|---|---|---|
| | female CVD mortality 2017 | | male CVD mortality 2017 | |
| | (1) | (2) | (3) | (4) |
| constant | 27.10 (37.18) | -118.15 (91.70) | 99.23 (50.56) | -181.55 (105.32) |
| GSNI2 | 3.57*** (0.55) | 4.27*** (0.85) | 3.59*** (0.74) | 4.78*** (0.98) |
| physicians per 1000 | | 8.39 (15.71) | | 39.43* (18.04) |
| mean years of schooling | | 20.53* (8.80) | | 29.30** (10.10) |
| GDP per capita | | -0.002* (0.001) | | -0.004*** (0.001) |
| GSNI data collection period 2010-2014 | | -90.81* (38.64) | | -115.70* (44.37) |
| $R^2$ | 0.37 | 0.49 | 0.24 | 0.55 |
| Adjusted $R^2$ | 0.36 | 0.45 | 0.23 | 0.51 |
| F Statistic | 42.72*** (df = 1; 73) | 11.80*** (df = 5; 61) | 23.38*** (df = 1; 73) | 14.80*** (df = 5; 61) |

* p<0.05, **p<0.01,***p<0.001

**Table S3.** Results of the sensitivity analysis univariable and multivariable regression models for the female and male CVD mortality outcomes. Numbers in brackets are standard errors. GSNI2 – Gender Social Norms Index 2; GDP – Gross Domestic Product; CVD – Cardiovascular disease

For peer review only

| | *Dependent variable:* | | | |
|---|---|---|---|---|
| | CVD mortality 2017 | | female to male CVD mortality ratio 2017 | |
| | (1) | (2) | (3) | (4) |
| constant | 60.45 (41.48) | -132.22 (93.98) | 0.58*** (0.05) | 0.92*** (0.10) |
| GSNI2 | 3.54*** (0.61) | 4.36*** (0.87) | 0.003*** (0.001) | 0.001 (0.001) |
| physicians per 1000 | | 22.02 (16.10) | | -0.04* (0.02) |
| mean years of schooling | | 23.92* (9.01) | | -0.01 (0.01) |
| GDP per capita | | -0.003** (0.001) | | -0.0000 (0.0000) |
| GSNI data collection period 2010-2014 | | -101.63* (39.59) | | -0.03 (0.04) |
| $R^2$ | 0.32 | 0.53 | 0.22 | 0.44 |
| Adjusted $R^2$ | 0.31 | 0.49 | 0.21 | 0.39 |
| F Statistic | 33.75*** (df = 1; 73) | 13.60*** (df = 5; 61) | 20.31*** (df = 1; 73) | 9.38*** (df = 5; 60) |

* p<0.05, **p<0.01,***p<0.001

**Table S4.** Results of the sensitivity analysis univariable and multivariable regression models for CVD mortality and female to male CVD mortaliy ratio outcomes Numbers in brackets are standard errors.
GSNI2 – Gender Social Norms Index 2; GDP – Gross Domestic Product; CVD – Cardiovascular disease

| | *Dependent variable:* | | | |
|---|---|---|---|---|
| | female life expectancy 2019 | | male life expectancy 2019 | |
| | (1) | (2) | (3) | (4) |
| constant | 87.73*** (1.36) | 60.75*** (4.23) | 82.27*** (1.42) | 38.63*** (5.06) |
| GSNI | -0.15*** (0.02) | -0.04* (0.02) | -0.15*** (0.02) | -0.02 (0.02) |
| log (physicians per 1000) | | 0.92** (0.28) | | 0.81* (0.36) |
| mean years of schooling | | -0.29 (0.17) | | -0.47 (0.23) |
| log (GDP per capita) | | 2.46*** (0.43) | | 4.28*** (0.56) |
| GSNI data collection period 2010-2014 | | 1.32 (0.78) | | 1.12 (1.06) |
| MMR | | -0.01*** (0.003) | | |
| $R^2$ | 0.45 | 0.88 | 0.40 | 0.77 |
| Adjusted $R^2$ | 0.44 | 0.87 | 0.39 | 0.75 |
| F Statistic | 58.99*** (df = 1; 73) | 70.88*** (df = 6; 59) | 48.54*** (df = 1; 73) | 40.03*** (df = 5; 61) |

* p<0.05, **p<0.01,***p<0.001

**Table S5.** Results of the sensitivity analysis univariable and multivariable regression models for female and male life expectancy at birth. Numbers in brackets are standard errors. GSNI2 – Gender Social Norms Index 2; GDP – Gross Domestic Product; CVD – Cardiovascular disease; MMR – Maternal mortality ratio

For peer review only

STROBE Statement—checklist of items that should be included in reports of observational studies

| | Item No | Recommendation | Page No |
|---|---|---|---|
| **Title and abstract** | 1 | (*a*) Indicate the study's design with a commonly used term in the title or the abstract | 1 |
| | | (*b*) Provide in the abstract an informative and balanced summary of what was done and what was found | 4 |
| **Introduction** | | | |
| Background/rationale | 2 | Explain the scientific background and rationale for the investigation being reported | 5 |
| Objectives | 3 | State specific objectives, including any prespecified hypotheses | 5 |
| **Methods** | | | |
| Study design | 4 | Present key elements of study design early in the paper | 6 |
| Setting | 5 | Describe the setting, locations, and relevant dates, including periods of recruitment, exposure, follow-up, and data collection | 6 |
| Participants | 6 | (*a*) *Cohort study*—Give the eligibility criteria, and the sources and methods of selection of participants. Describe methods of follow-up *Case-control study*—Give the eligibility criteria, and the sources and methods of case ascertainment and control selection. Give the rationale for the choice of cases and controls *Cross-sectional study*—Give the eligibility criteria, and the sources and methods of selection of participants | 6 |
| | | (*b*) *Cohort study*—For matched studies, give matching criteria and number of exposed and unexposed *Case-control study*—For matched studies, give matching criteria and the number of controls per case | NA |
| Variables | 7 | Clearly define all outcomes, exposures, predictors, potential confounders, and effect modifiers. Give diagnostic criteria, if applicable | 6-7 |
| Data sources/ measurement | 8* | For each variable of interest, give sources of data and details of methods of assessment (measurement). Describe comparability of assessment methods if there is more than one group | 6-7 |
| Bias | 9 | Describe any efforts to address potential sources of bias | 6-8 |
| Study size | 10 | Explain how the study size was arrived at | NA |
| Quantitative variables | 11 | Explain how quantitative variables were handled in the analyses. If applicable, describe which groupings were chosen and why | 7-8 |
| Statistical methods | 12 | (*a*) Describe all statistical methods, including those used to control for confounding | 7-8 |
| | | (*b*) Describe any methods used to examine subgroups and interactions | NA |
| | | (*c*) Explain how missing data were addressed | 7-8 |
| | | (*d*) *Cohort study*—If applicable, explain how loss to follow-up was addressed *Case-control study*—If applicable, explain how matching of cases and controls was addressed *Cross-sectional study*—If applicable, describe analytical methods taking account of sampling strategy | NA |
| | | (*e*) Describe any sensitivity analyses | 7-8 |

Continued on next page



For peer review only - http://bmjopen.bmj.com/site/about/guidelines.xhtml

| | | | |
|---|---|---|---|
| **Results** | | | |
| Participants | 13* | (a) Report numbers of individuals at each stage of study—eg numbers potentially eligible, examined for eligibility, confirmed eligible, included in the study, completing follow-up, and analysed | NA |
| | | (b) Give reasons for non-participation at each stage | NA |
| | | (c) Consider use of a flow diagram | NA |
| Descriptive data | 14* | (a) Give characteristics of study participants (eg demographic, clinical, social) and information on exposures and potential confounders | 15-16 |
| | | (b) Indicate number of participants with missing data for each variable of interest | 15-16 |
| | | (c) *Cohort study*—Summarise follow-up time (eg, average and total amount) | NA |
| Outcome data | 15* | *Cohort study*—Report numbers of outcome events or summary measures over time | NA |
| | | *Case-control study*—Report numbers in each exposure category, or summary measures of exposure | NA |
| | | *Cross-sectional study*—Report numbers of outcome events or summary measures | 15-16 |
| Main results | 16 | (*a*) Give unadjusted estimates and, if applicable, confounder-adjusted estimates and their precision (eg, 95% confidence interval). Make clear which confounders were adjusted for and why they were included | 18-20 |
| | | (*b*) Report category boundaries when continuous variables were categorized | NA |
| | | (*c*) If relevant, consider translating estimates of relative risk into absolute risk for a meaningful time period | NA |
| Other analyses | 17 | Report other analyses done—eg analyses of subgroups and interactions, and sensitivity analyses | 10 |
| **Discussion** | | | |
| Key results | 18 | Summarise key results with reference to study objectives | 10 |
| Limitations | 19 | Discuss limitations of the study, taking into account sources of potential bias or imprecision. Discuss both direction and magnitude of any potential bias | 11-12 |
| Interpretation | 20 | Give a cautious overall interpretation of results considering objectives, limitations, multiplicity of analyses, results from similar studies, and other relevant evidence | 12-14 |
| Generalisability | 21 | Discuss the generalisability (external validity) of the study results | 11 |
| **Other information** | | | |
| Funding | 22 | Give the source of funding and the role of the funders for the present study and, if applicable, for the original study on which the present article is based | 23 |

*Give information separately for cases and controls in case-control studies and, if applicable, for exposed and unexposed groups in cohort and cross-sectional studies.

**Note:** An Explanation and Elaboration article discusses each checklist item and gives methodological background and published examples of transparent reporting. The STROBE checklist is best used in conjunction with this article (freely available on the Web sites of PLoS Medicine at http://www.plosmedicine.org/, Annals of Internal Medicine at http://www.annals.org/, and Epidemiology at http://www.epidem.com/). Information on the STROBE Initiative is available at www.strobe-statement.org.



For peer review only - http://bmjopen.bmj.com/site/about/guidelines.xhtml
